# An enhanced respiratory mechanics model based on double-exponential and fractional calculus

**DOI:** 10.3389/fphys.2023.1273645

**Published:** 2023-12-04

**Authors:** Zongwei Li, Yanbin Pei, Yuqi Wang, Qing Tian

**Affiliations:** ^1^ Department of Thoracic Surgery, The First Medical Centre, Chinese PLA General Hospital, Beijing, China; ^2^ Medical School of Chinese PLA, Beijing, China

**Keywords:** double-exponential model, fractional calculus, machine learning, mechanical ventilation, respiratory mechanics models

## Abstract

We address mathematical modelling of respiratory mechanics and put forward a model based on double-exponential and fractional calculus for parameter estimation, model simulation, and evaluation based on actual data. Our model has been implemented on a publicly available executable code with adjustable parameters, making it suitable for different applications. Our analysis represents the first application of fractional calculus and double-exponential modelling to respiratory mechanics, and allows us to propose a hybrid model fitting experimental data in different ventilation modes. Furthermore, our model can be used to study the mechanical features of the respiratory system, improve the safety of ventilation techniques, reduce ventilation damages, and provide strong support for fast and adaptive determination of ventilation parameters.

## 1 Introduction

With the aging population and the increase in chronic respiratory diseases (Divo et al., 2014; Soriano et al., 2020; Xie et al., 2020), the demand for mechanical ventilation treatment has largely increased (Wunsch, 2020). At the same time, the outbreak of COVID-19 in recent years has further boosted the demand for ventilators. As a consequence, the analysis of more accurate respiratory mechanics models has become extremely important, as it can be used to predict and optimize parameter settings in mechanical ventilation therapy. Further, this kind of models are required to dynamically adjust parameters according to the respiratory characteristics of different patients, in order to improve treatment outcomes and reduce ventilation injuries.

In the field of mechanical ventilation, the respiratory system is often modelled as a linear dynamical system described by linear differential equations. Examples are provided by the single-compartment model (Bates, 2009; Ghafarian et al., 2016; Gertler, 2021) corresponding to a first-order linear differential system, the two-compartment model (Bates, 2009; Carvalho and Zin, 2011; Ghafarian et al., 2016) corresponding to a second-order differential system, and the viscoelastic model (Ganzert et al., 2009). Over the last few years, different types of respiratory mechanics models have been put forward, including nonlinear models involving elastance and resistance of the respiratory system (Chiew et al., 2015; Redmond et al., 2017; Ellwein Fix et al., 2018; Marconi and De Lazzari, 2020). These studies generally provide a good theoretical modelling and may be used to simulate the respiratory system. However, they generally involve idealized elementary function description of ventilation pressure (Ellwein Fix et al., 2018; Marconi and De Lazzari, 2020; Dincel, 2021), and ignore the inherent randomness and background noise/error of real respiratory processes. Other studies are based on experimental data, but produce results in disagreement with actual data. For example, the simulations of Albanese et al. (2016), lead to a minimum CV(RMSE) (stands for the coefficient of variations of the root mean square errors) volume curve equal to 0.079, which is an order of magnitude higher than the simulation results of all models in this paper. At the same time, these studies did not provide executable program code and corresponding original data.

In this paper, we propose an accurate respiratory mechanics model based on previous research and studies based on actual measurement data. We consider Bama pigs for actual measurements, since they provide conditions close to the healthy state of the human respiratory system, and assess results of our models against actual measurement data. By combining fractional calculus with a double-exponential model, we are able to describe the dynamical behaviour of the coefficients in the differential equations, and to put forward a hybrid model consistent with the experimental in wide range of ventilation modes, and provided. We also provide a public executable code package for parameter estimation, simulation methods, and optimized parameter search.

## 2 Methods

### 2.1 Respiratory mechanics models

#### 2.1.1 Linear models

First order linear differential models with constant coefficients (linear single-compartment model). According to previous studies on respiratory mechanics models (Bates, 2009; Carvalho and Zin, 2011; Hess, 2014; Ghafarian et al., 2016; Gertler, 2021), the classical linear single-compartment model corresponds to the following differential equation:

$$P\left(t\right)=R\frac{dV\left(t\right)}{dt}+EV\left(t\right)+P_{0}$$ (1)

for the pressure (applied power) to the lung P(t), where V(t) is the air volume entering the lung (Tidal volume), E and R denote the elastance and resistance of respiratory system respectively. The offset pressure P_0_ represents the positive end-expiratory pressure (PEEP). The compliance C of respiratory system is the reciprocal of elastance E, that is C = 1/E.

**FIGURE 1:**
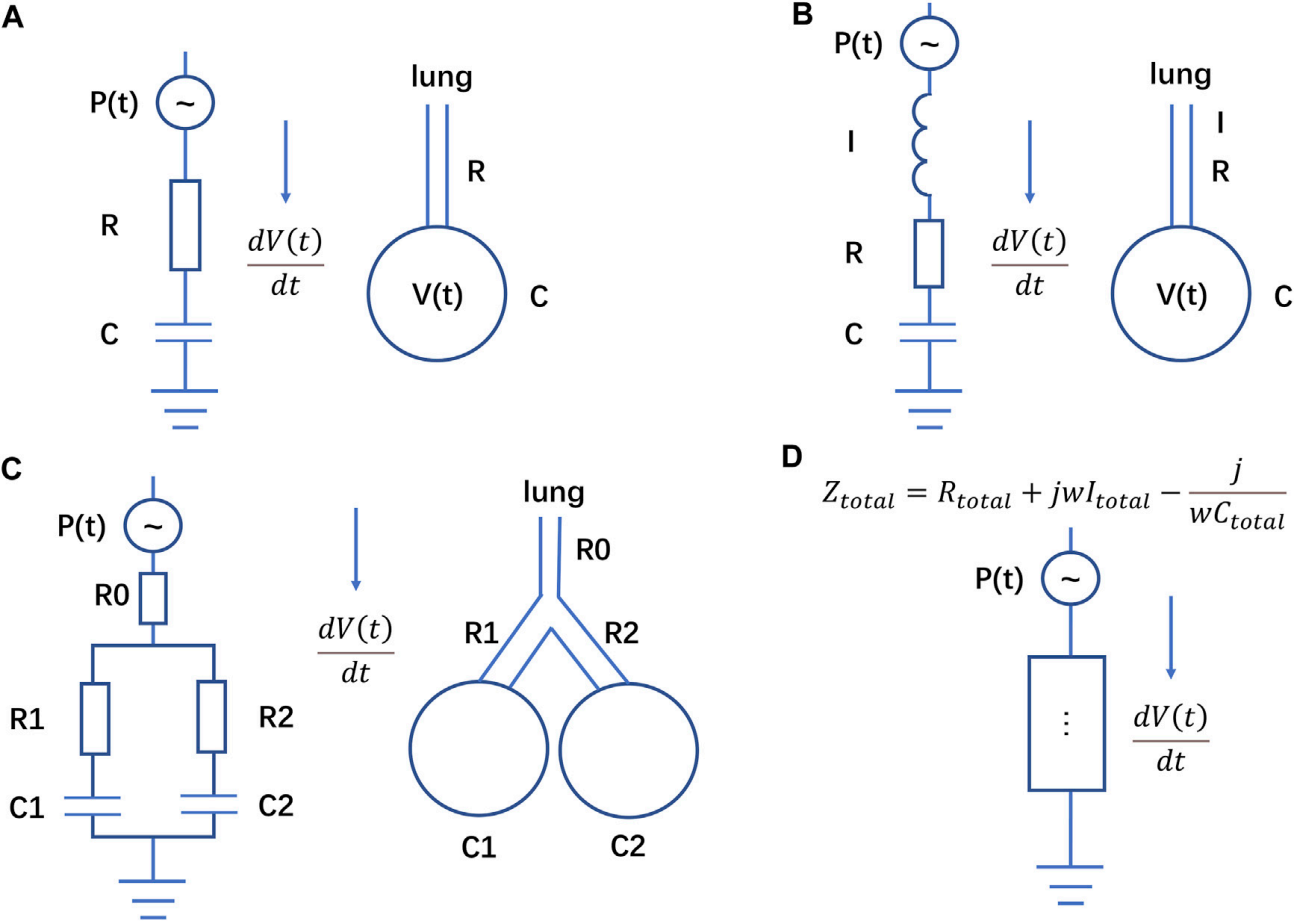
Electrical analogue of the respiratory system. **(A)** Scheme corresponding to Eq. 1, **(B)** scheme corresponding to Eq. 10, **(C)** scheme corresponding to Eqs 14, **(D)** different impedance elements in series and in parallel corresponding to Eq. 16. The resistance R of respiratory system in Eq. 1 is equivalent to the resistance in the circuit, and the compliance C of respiratory system is equivalent to the capacitor in the circuit. The current in the circuit represents the flow in the airway.

The electrical circuit that represents the linear single-compartment model consists of a resistance R, a capacitor C (1/E) and a generator P(t) (Figure 1A). The corresponding impedance is (Carvalho and Zin, 2011; Li, 2011):

$$Z=R-\frac{j}{\omega C}$$ (2)

where ω is the angular frequency in radians/second, and the symbol j represents the unit imaginary number.

Moreover, based on the electrical analogous (a), if we assume the initial value V (0) = 0 and P_0_ = 0 (or define a new pressure ${\hat{P}\left(t\right)=P\left(t\right)-P}_{0}$ ) for simplicity, we can obtain the open-loop transfer function between the pressure P(t) and volume V(t):

$$G\left(s\right)=\frac{V\left(s\right)}{P\left(s\right)}=\frac{C}{CRs+1}=\frac{1}{Rs+E}$$ (3)

The inverse Laplace transform of transfer function G(s) is given by:

$${G\left(t\right)=L}^{-1}\left(G\left(s\right)\right)=L^{-1}\left(\frac{\frac{1}{R}}{s+\frac{1}{CR}}\right)=\frac{1}{R}e^{-\frac{t}{CR}}=\frac{1}{R}e^{-\frac{tE}{R}}$$ (4)

If P(t) is a unit step signal, this will be the solution in time domain.

If we express the pressure P(t) as a linear combination of elementary functions:

$$P\left(t\right)=\sum_{i=0}^{n}{k_{i}P}_{i}\left(t\right)$$ (5)

Such as sin (at), $e^{-at}$ , or polynomial functions, and the k_i_ are constant coefficients, then we can theoretically obtain the general solution of V(t) in time domain as follows

$$V\left(t\right)=L^{-1}\left(G\left(s\right)P\left(s\right)\right)=L^{-1}\left(G\left(s\right)\right)*L^{-1}\left(P\left(s\right)\right)=\int_{0}^{t}G\left(t-\tau \right)P\left(\tau \right)d\tau$$ (6)

where “*” denotes convolution. The result is the same as that obtained by directly solving the equation by Laplace transform. Based on the superposition principle of linear differential equation, the final solution of V(t) is given by

$$V\left(t\right)=\sum_{i=0}^{n}\int_{0}^{t}k_{i}G\left(t-\tau \right)P_{i}\left(\tau \right)d\tau$$ (7)

For example, if ${P\left(t\right)=P}_{constant}\sin\left(\text{at}\right),t\in \left[0,\pi \right]$ , Eq. 1 can be written as: $R\frac{dV\left(t\right)}{dt}+EV\left(t\right)=P_{constant}\sin\left(\text{at}\right)-P_{0}$ , with general solution $V\left(t\right)=Ke^{-\frac{t}{CR}}+\frac{PC}{a^{2}C^{2}R^{2}+1}\left(\sin\left(at\right)-aCRcos\left(at\right)\right)-CP_{0}$ , where K is a constant determined by the initial conditions and P_constant_ is also a constant that determines the magnitude of pressure. Other simple examples are illustrated in the Supplementary Material Section S1.

Although it is theoretically possible to represent any P(t) based on the Fourier transform, this method is not particularly useful due to the computational complexity and the lack of accuracy.

Second order models with constant coefficients. Assuming that the airway is an approximately cylindrical structure of length l and radius r and using Newton’s second law (Carvalho and Zin, 2011; Hess, 2014) we have:

$$\pi r^{2}P_{i}=P_{i}S=F=ma=\pi r^{2}l\rho \left(\frac{d^{2}V}{dt^{2}}\right)$$ (8)

that is:

$$P_{i}=l\rho \frac{d^{2}V}{dt^{2}}=I\frac{d^{2}V}{dt^{2}}$$ (9)

where ρ is the gas density, and I=lρ . Due to the relatively low gas density, the inertia coefficient I (Inertia) is generally very small compared to other parameters with the same dimensions in respiratory mechanics models. Taking airway resistance and elasticity into account, the pressure P and volume V are related by the following Eq. 10.

$$P\left(t\right)=I\frac{d^{2}V\left(t\right)}{dt^{2}}+R\frac{dV\left(t\right)}{dt}+EV\left(t\right)+P_{0}$$ (10)

This is a second order linear differential equation, which corresponds to the electrical analogue shown in Figure 1B with impedance:

$$Z=R+j\omega I-\frac{j}{\omega C}$$ (11)

where I represents the total inductance of the circuit.

If we assume the initial conditions V (0) = 0 and V’ (0) = 0 (the first-order derivative of V) and P_0_ = 0 (or define new pressure ${\hat{P}\left(t\right)=P\left(t\right)-P}_{0}$ ) for simplicity, we can also obtain the transfer function, which can be written as the sum of two fractions:

$$G\left(s\right)=\frac{V\left(s\right)}{P\left(s\right)}=\frac{1}{Is^{2}+Rs+E}=\frac{c_{1}}{s+a}+\frac{c_{2}}{s+b}$$ (12)

where a, b, c_1_, c_2_ are constant and the real part of a and b are positive, that is Re(a) > 0, Re(b) > 0 (satisfying the stability constraint conditions) (Zhong, 1988; Hu, 2007).

The inverse Laplace transform of the transfer function in Eq. 12 is given by:

$$G\left(t\right)=L^{-1}\left(G\left(s\right)\right)=L^{-1}\left(\frac{c_{1}}{s+a}+\frac{c_{2}}{s+b}\right)=c_{1}e^{-at}+c_{2}e^{-bt}$$ (13)

Assuming that P(t) is a step signal, the general solution of Eq. 10 has the same form as Eq. 14. On the other hand, if we have single-compartment models connected in series or in parallel, we obtain the two-compartment model proposed by Bates (Bates, 2009), whose dynamics is similar to that governed by Eq. 10 (Figure 1C), i.e.,

$$P\left(t\right)+k\frac{dP\left(t\right)}{dt}=I\frac{d^{2}V\left(t\right)}{dt^{2}}+R\frac{dV\left(t\right)}{dt}+EV\left(t\right)$$ (14)

where k is a constant. The transfer function of Eq. 14 is similar to that reported in Eq. 12:

$$G\left(s\right)=\frac{V\left(s\right)}{P\left(s\right)}=\frac{1+ks}{Is^{2}+Rs+E}=\frac{k_{1}}{s+a}+\frac{k_{2}}{s+b}$$ (15)

In other words, Eqs 10, 14 has the same solutions.

According to the superposition principle, we can also obtain the general solution of second order linear differential models based on Eq. 7. Simple examples are given in the Supplementary Material Section 2.

High-order linear differential models. The term $I\frac{d^{2}V\left(t\right)}{dt^{2}}$ in Eq. 10 corresponds to an acceleration, however, in a respiratory mechanical model (Carvalho and Zin, 2011; Eager et al., 2016; Dincel, 2021) we may also consider higher derivatives (like jerk and snap), such that more general models may be obtained, in the following general form:

$$b_{0}P\left(t\right){+b}_{1}\frac{dP\left(t\right)}{dt}+\ldots +b_{m}\frac{d^{m}P\left(t\right)}{dt^{m}}=a_{0}V\left(t\right)+a_{1}\frac{dV\left(t\right)}{dt}+a_{2}\frac{d^{2}V\left(t\right)}{dt^{2}}+\ldots +a_{n}\frac{d^{n}V\left(t\right)}{dt^{n}}$$ (16)

where n > m.

Assuming the initial values V (0), V’ (0), V’’ (0), … = 0, we obtain the transfer function as

$$G\left(s\right)=\frac{V\left(s\right)}{P\left(s\right)}=\frac{b_{m}s^{m}+\ldots +b_{2}s^{2}+b_{1}s+b_{0}}{a_{n}s^{n}+\ldots +a_{2}s^{2}+a_{1}s+a_{0}}$$ (17)

In a stable respiratory system, the system should satisfy Routh-Hurwitz criterion. The inverse Laplace transform of transfer function G(s) is as follows:

$${G\left(t\right)=L}^{-1}\left(G\left(s\right)\right)=\sum_{i=0}^{n}{k_{i}e}^{\lambda_{i}t}$$ (18)

where ${\text{Re}\left(\lambda \right.}_{i})<0$ .

If P(t) is a unit step signal, this represents the solution of Eq. 16.

Using again the electrical analogy, Eq. 16 corresponds to put more components in series or in parallel (in a circuit, components connected in parallel are equivalent to adding their transfer functions in the s domain, while components connected in series are equivalent to multiplying their transfer functions in the s domain). The overall circuit has an equivalent impedance Z_total_:

$$Z_{total}=R_{total}+j\omega I_{total}-\frac{j}{\omega C_{total}}$$ (19)

where R_total_, I_total_, C_total_ represent the overall equivalent resistance, capacitor, inductance respectively in the circuit (Figure 1D). Eq. 19 and Eq. 11 have indeed a similar form.

However, high-order linear differential models are rarely encountered and applied in practical applications. We suggest caution in using high-order models to describe the respiratory system, primarily based on the following reasons:

1. Since Eq. 19 and Eq. 11 have a similar form, we can approximate the effect of high-order differential models by using first and second order differential models.
2. Higher-order differential models are difficult to stabilize in practical applications, as there are always disturbances present in real-world situations. We illustrate some examples in the Supplementary Material Section 3.
3. There are many parameters in high-order differential models, which typically require more measured data to be estimated. In the following sections, we introduce fractional calculus models with fewer parameters and similar accuracy.
4. In nondegenerate mechanics systems, (the Hessian matrix is full rank), based the Hamilton’s Principle and Ostrogradsky’s theorem, terms of order three and higher in higher-order differential models do not exist. More details on thus point may be found in the Supplementary Material Section 4.

Overall, given the above analysis, we are not going to employ high-order differential models in processing measured data.

#### 2.1.2 Fractional calculus models

If there is uncertainty about the appropriate order of the differential terms in the respiratory mechanics model and on the actual weight of each order, we may consider introducing fractional-order terms involving different orders of derivatives into the model. This would also allow us to take into account the memory-dependent viscoelasticity and heterogeneity features of the lung tissue (Bates, 2009; Ganzert et al., 2009; Chen et al., 2010; Carvalho and Zin, 2011; Ionescu and Kelly, 2017; Xue and Bai, 2023), which cannot be captured by classical integer-order differential equations-based lumped-parameter models. Here, we propose a fractional calculus model to solve this problem, which has fewer parameters to be estimated compared to classical models.

Fractional calculus may be introduced in different and slightly inequivalent ways, involving Grünwald-Letnikov, Riemann–Liouville, and Caputo fractional derivatives. Here, we primarily consider the Grünwald-Letnikov definition that is convenient for numerical calculations. The α-order derivative of a given function f(t) is expressed as (Xue and Bai, 2023):

$$D_{t_{0}}^{\alpha }f\left(t\right)=\lim_{h\to 0}\frac{1}{h^{\alpha }}\sum_{j=0}^{\frac{t-t_{0}}{h}}\frac{{\left(-1\right)}^{j}\Gamma \left(\alpha +1\right)}{\Gamma \left(j+1\right)\Gamma \left(\alpha -j+1\right)}f\left(t-jh\right)$$ (20)

In the above formula, h is a sufficiently small time step, and Γ denotes the Gamma function, that is $\Gamma \left(z\right)=\int_{0}^{\infty }e^{-t}t^{z-1}dt$ 。This definition assumes that the value of the function f(t) is 0 when t ≤ *t*_0_, in agreement with the assumption that any physical quantity is zero before the initial time t0. Fractional order derivatives are able to capture memory properties (applicable to differentiation where α > 0 and to integration where α < 0). More about numerical calculations of fractional order calculus can be found in the Supplementary Material Section 5.

Furthermore, according to Eq. 20, any fractional order derivative term also involves some higher order derivative features.

Based on the fractional order differentiation proposed above, we modify Eqs 1, 20, thus obtaining the following fractional order derivative respiratory mechanics model:

$$P=EV\left(t\right)+RV^{\prime }\left(t\right)+aD_{t_{0}}^{\alpha }V\left(t\right)+P_{0}$$ (21)

where $D_{t_{0}}^{\alpha }V\left(t\right)$ is the α-order derivative of the $V\left(t\right)$ , with a fractional order differential term α > 0. When α = 1, it takes the form of Eq. 10. In the subsequent sections, we mainly focus on cases with 0<α < 1.

The above formula contains a single fractional order derivative term. Analogously, we may also introduce models containing more fractional terms, such as the generalization of Eq. 22 containing 2 fractional order derivative terms, i.e.,

$$P=EV\left(t\right)+RV^{\prime }\left(t\right)+aD_{t_{0}}^{\alpha }V\left(t\right)+bD_{t_{0}}^{\beta }V\left(t\right)+P_{0}$$ (22)

As a matter of fact, upon considering actual measurement data, one sees that more fractional order terms do not provide better results than a single one. Parameters estimation and model simulation results for Eq. 21, 22 are reported in the Supplementary Material Section 6.

#### 2.1.3 Non-linear models

The models mentioned earlier are all cases where the coefficients of the differential equations are constants. In the actual respiratory system, the elasticity E and airway resistance R may be variables rather than constants (Van Drunen et al., 2014; Chiew et al., 2015; Redmond et al., 2017; Ellwein Fix et al., 2018; Marconi and De Lazzari, 2020). When the elasticity E changes with V(t), and the resistance R changes with V′(t), based on Eq. 1, pressure P(t) and volume V(t) satisfy the following equation:

$$P\left(t\right)=Ef\left(V\left(t\right)\right)V\left(t\right)+Rh\left(V^{\prime }\left(t\right)\right)V^{\prime }\left(t\right)+P_{0}$$ (23)

where V′(t) represents the first-order derivative of V with respect to t.

Specifically, when only the elasticity E varies with V(t) or only the resistance R varies with V′(t), Eq. 23 can be evolved into Eq. 24 and Eq. 25.

$$P\left(t\right)=Ef\left(V\left(t\right)\right)V\left(t\right)+RV^{\prime }\left(t\right)+P_{0}$$ (24)

$$P\left(t\right)=EV\left(t\right)+Rh\left(V^{\prime }\left(t\right)\right)V^{\prime }\left(t\right)+P_{0}$$ (25)

Nonlinear models containing polynomial terms. In order to describe physical situations in which the elasticity E is monotonically (and strongly) positively correlated with V(t) and the resistance R is monotonically strongly positively correlated with V′(t), we should consider polynomial functions f and h in Eq. 23. To a first approximation, we may safely ignore the different inflection points of the polynomials and focus on the highest order term that mostly affects the behaviour of the functions. This amounts to consider f of the form of $E_{offset}+\alpha {V\left(t\right)}^{\beta }$ , where E_offset_ > 0, and the constants α and β capture the behaviour of the elasticity E as a function of V(t). The form of the function h is analogue, i.e., $R_{offset}+\alpha {V^{\prime }\left(t\right)}^{\beta }$ . Overall, we have

$$P\left(t\right)=E_{0}\left(E_{offset}+\alpha_{E}{V\left(t\right)}^{\beta_{E}}\right)V\left(t\right)+R_{0}\left(R_{offset}+\alpha_{R}{V^{\prime \left(t\right)}}^{\beta_{R}}\right)V^{\prime }\left(t\right)+P_{0}$$ (26)

Then Eq. 23 can be written as

$$P\left(t\right)=EV\left(t\right)+RV^{\prime }\left(t\right)+a{V\left(t\right)}^{x}+b{V^{\prime \left(t\right)}}^{y}+P_{0}$$ (27)

where a, b, x and y are constant.

Similarly, when only the elasticity E changes with V(t) in a polynomial form or only the resistance R changes with V′(t) in a polynomial form, Eq. 27 can be written as

$$P\left(t\right)=EV\left(t\right)+RV^{\prime }\left(t\right)+a{V\left(t\right)}^{x}+P_{0}$$ (28)

$$P\left(t\right)=EV\left(t\right)+RV^{\prime }\left(t\right)+b{V^{\prime \left(t\right)}}^{y}+P_{0}$$ (29)

As a matter of fact, there exists some nonlinear models with analytic solutions in terms of elementary functions. For instance, for the model $\left(t\right)=E_{0}\left(E_{offset}+kV\left(t\right)\right)V\left(t\right)+RV^{\prime }\left(t\right)+P_{0}=EV\left(t\right)+RV^{\prime }\left(t\right)+a{V\left(t\right)}^{2}+P_{0}$ , if we assume the P(t) is a step signal with constant value P_c_, then its analytic general solution is $V\left(t\right)=\frac{-E+\sqrt{-E^{2}+4a\left(P_{0}-P_{c}\right)}\tan\left[\frac{\sqrt{-E^{2}+4a\left(P_{0}-P_{c}\right)}\left(-t+kR\right)}{2R}\right]}{2a}$ , where k is the constant coefficient determined by the initial conditions.

Non-linear models containing exponential terms. In some cases, the respiratory system elasticity E may show a step response property^10,12^. Combined with the results presented in the following sections, this corresponds to the model described by Eq. 24. If function f is an exponential function, double-exponential function and triple exponential function, P and V satisfy one of the following equations.

$$P\left(t\right)=EV\left(t\right)+ae^{xV\left(t\right)}V\left(t\right)+RV^{\prime }\left(t\right)+P_{0}$$ (30)

$$P\left(t\right)=EV\left(t\right)+a{e^{e}}^{xV\left(t\right)}V\left(t\right)+RV^{\prime }\left(t\right)+P_{0}$$ (31)

$$P\left(t\right)=EV\left(t\right)+a{e^{e^{e}}}^{xV\left(t\right)}V\left(t\right)+RV^{\prime }\left(t\right)+P_{0}$$ (32)

The growth in single exponential function is fast, but not as fast as the factorial function. The double-exponential function grows faster than the factorial function (see the Supplementary Material Section 7). The growth rate of the Ackermann’s function is much faster than the double-exponential function. As it may intuitively be expected, we will show that without using piecewise functions the faster is the growth, the better is the fitting of the measured data (for a step response).

#### 2.1.4 Hybrid model

Considering the step-like features of the respiratory system elasticity, as well as the memory-dependent viscoelasticity and heterogeneity characteristics of lung tissue, we may combine Eq. 21 with Eq. 30 to obtain the hybrid model of Eq. 33, which itself incorporates different properties:

$$P\left(t\right)=EV\left(t\right)+RV^{\prime }\left(t\right)+aD_{t_{0}}^{\alpha }V\left(t\right)+b{e^{e}}^{\beta V\left(t\right)}+P_{0}$$ (33)

As we will see in the following Sections, this hybrid model provides the best fit for measured data in most situations.

### 2.2 Data collection

We use a Dräger Savina 300 to measure airway pressure P(t), flow speed V’(t) and respiratory volume V(t) in the actual respiration of three Bama pigs. The first Bama pig (sample ID as case1) weighs 28 kg, and data are taken for three ventilation modes, namely, pressure control - assist control (PC-AC), volume control - assist control (VC-AC), and volume control - synchronized intermittent mandatory ventilation (VC-SIMV). The second one (sample ID as case2) weighs 31 kg and the third one (sample ID as case3) weighs 24.3 kg, and data are taken for the VC-AC ventilation mode. In all measurements, pressure P(t), flow speed V′(t), and respiratory volume V(t) data are collected every 10 ms for at least 6 min. In the pressure control ventilation mode, set peak breathing pressure = 16 mbar, plateau pressure = 15 mbar and PEEP = 3 mbar. In the volume control ventilation mode, the tidal volume is set to 0.3 L.

During the measurement process, the original HL7 data are first obtained through serial communication, and then the pressure P, flow speed V′, and respiratory volume V data at each moment are further parsed and extracted. The data at the same moment are placed in the same row, with each row divided into three columns: pressure P, flow speed V’, and respiratory volume V, separated by tabs. The data are arranged in chronological order. Finally, the unit of P is converted to mbar, the flow rate is in L/s, and the respiratory volume is in L, which are written into the txt file.

For cross-validation at different time periods, two data files of 150 s duration (15,000 data points, about 50 breathing cycles) are extracted from each breathing mode. After sorting, the data files are respectively named as case1PCAC1.txt, case1VCAC1.txt, case1VCSIMV1.txt, case2VCAC1.txt, case3VCAC1.txt and case1PCAC2.txt, case1VCAC2.txt, case1VCSIMV2.txt, case2VCAC2.txt case3VCAC2.txt.

### 2.3 Parameter estimation

Parameter estimation for all the above-mentioned ordinary differential equations with constant coefficients can be performed using the following procedure (Bates, 2009; Redmond et al., 2017; Avilés-Rojas and Hurtado, 2022). The first observation is that all the models analyzed in this paper may be recasted as

$$P\left(t\right)=a_{1}f_{1}\left(t\right)+a_{2}f_{2}\left(t\right)+\ldots +a_{m}f_{m}\left(t\right)+P_{0}$$ (34)

where $\left\{f_{1}\left(t\right),f_{2}\left(t\right)\ldots f_{n}\left(t\right)\right\}$ denotes any linear or nonlinear term of the differential equation, for example, $f_{n}\left(t\right)$ may represent ${\left(V^{\prime }\left(t\right)\right)}^{2}$ , $D_{t_{0}}^{\alpha }V\left(t\right)$ , $e^{xV\left(t\right)}$ , etc., while $\vec{A}=\left\{a_{1},a_{2}\ldots a_{n}\right\}$ is the corresponding set of coefficients, i.e., the quantities to be estimated.

The values of pressure P(t), flow speed V′(t), and respiratory volume V(t) collected in real time are organized in vector and matrix form and used as input for the algorithm. If the collected values of the respiratory system are at n time points $\left\{t_{1},t_{2}\ldots t_{n}\right\}$ , then the input data are the n-dimensional pressure vector P(t), and the data matrix X with n rows and m+1 columns:

$$\vec{P}=\left\{\begin{array}{c} \left.{P(t}_{1}\right)\\ \left.{P(t}_{2}\right)\\ \begin{array}{c} \vdots \\ \left.{P(t}_{n}\right) \end{array} \end{array}\right\}$$ (35)

$$X=\left\{\begin{array}{c} \begin{array}{ccc} f_{1}\left(t_{1}\right) & \ldots & \begin{array}{cc} f_{m}\left(t_{1}\right) & 1 \end{array} \end{array}\\ \begin{array}{ccc} f_{1}\left(t_{2}\right) & \ldots & \begin{array}{cc} f_{m}\left(t_{2}\right) & 1 \end{array} \end{array}\\ \begin{array}{c} \vdots \\ \begin{array}{ccc} f_{1}\left(t_{n}\right) & \ldots & \begin{array}{cc} f_{m}\left(t_{n}\right) & 1 \end{array} \end{array} \end{array} \end{array}\right\}$$ (36)

Then, the coefficients can be estimated from

$$\vec{A}={\left[X^{T}X\right]}^{-1}X^{T}\vec{P}$$ (37)

In the above Eq. 37, $X^{T}$ represents matrix transposition, and ${\left[*\right]}^{-1}$ represents matrix inversion. We estimate parameters by minimizing the sum of squared residuals (SSR). At the extreme point, the partial derivatives with respect to each coefficient are zero, and this provides constraints to obtain their values. In order to determine the fractional-order in Eq. 21, we employ Particle Swarm Optimization (PSO) algorithm in machine learning (Kennedy and Eberhart, 1995; Gad, 2022; Shami et al., 2022; Xie et al., 2022; Yang et al., 2022), searching for the stable optimal solution with the lowest overall SSR. In this way, we also find the coefficient x for the exponential Eqs 30–32, and the hybrid Eq. 33. For all the models, suitable values of the number of particles N and iterations M may be found, which ensure stable optimal solutions. In general, for Eq. 21, M = N = 5 is enough, whereas for the exponential model we need M = N = 10, and for the hybrid model, M = N = 20. Once the values of α and x are determined, their corresponding constant coefficients can be calculated according to Eq. 37.

To ensure computational accuracy, we use the different packages in Python to calculate the fractional order derivatives (Adams, 2019), and call Python code within MATLAB while optimizing the SSR value of the models. In addition, since the exponential Eqs 30–32, is prone to produce a singular matrix, we perform address Eq. 37 using a pseudo-inverse matrix to reduce the distortion and deviation of numerical results.

More details about faster empirical estimation methods of the parameters for fractional calculus and exponential terms may be found in the Supplementary Material Section 8.

### 2.4 Numerical simulations

After obtaining the estimated values of the parameters, the stability of the models is assessed using the Routh-Hurwitz stability criterion, and then the subsequent simulations are carried out carefully considering their actual physical meaning (Hu, 2007; Wang et al., 2007). Generally, we accept estimated values of the parameters only if they are positive. On the one hand, this is required by the Routh-Hurwitz stability criterion, dictating that our models are stable only if all coefficients are positive. On the other hand, from a practical physical perspective, most of the variables of interest, e.g., the elasticity E of lung tissue are positive numbers.

After the parameters meet the above conditions, we use MATLAB/Simulink and Python to complete the calculations and construct the Simulink model for all the models considered in this work. The open-source vfoderiv3 module of Simulink (Scherer et al., 2011; Sierociuk et al., 2015; Sierociuk, 2023) is used for the simulation of fractional calculus. The Simulink model for the other models may be found in the Supplementary Material Section 9.

### 2.5 Evaluation criteria

The comparison of models with the same number of parameters to be estimated is made in terms of the SSR and the root mean square error (RMSE). Models with lower values of these two indicators should be preferred. The definition is given by

$$\text{SSR}=\sum_{i=1}^{n}{\left(V_{i}-\hat{V_{i}}\right)}^{2}$$ (38)

$$\text{RMSE}=\sqrt{\frac{SSR}{n}}$$ (39)

where $V_{i}$ represents the actual respiratory volume value from measured data at time i (the i th data point), and $\hat{V_{i}}$ represents the value from model simulations. The actual measurement period contains a total of n data points (an overall duration of 150 s corresponds to n = 15,000). The comparison between different models is made using the Bayesian Information Criterion (BIC) which considers the number of parameters. If $\text{residuals}∼N\left(0,\sigma^{2}\right)$ , we ignore the irrelevant constants and use the equivalent BIC value, i.e., Eq. 40, for model comparison and evaluation (Sheng et al., 2020).

$$\text{BIC}=n*\ln\left(\text{SSR}\right)+k*\ln\left(n\right)$$ (40)

In the above formula, k represents the number of model parameters. The process of deriving Eq. 40 based on the original BIC definition and the assumption of residual distribution may be found in the Supplementary Material Section 10.

In addition, we also use the Pearson correlation coefficient between the measured and simulated values of the respiratory volume as an additional criterion for optimization (the closer the correlation coefficient is to 1, the better).

### 2.6 Computation details

The executable code and actual respiratory data can be found in the Supplementary Material and our Gitee repository (https://gitee.com/lizwtest/mechanical_ventilation).

For models that do not contain exponential or fractional order differential terms, such as Eq 1 and Eq. 2 and Eq. 10, and (27–29), the measured respiratory mechanical data of the respiratory system can be substituted into Eq. 37 based on Section 2.3. This results in calculated values with practical physical characteristics such as respiratory system elasticity E, airway resistance R, P0 (PEEP), and so on.

For the calculation of models containing double exponential or fractional order differential terms, taking the double exponential model of Eq. 31 as an example, when a suitable value of the exponential parameter x is given in the model, the respiratory system mechanics data is substituted into Eq. 37 to obtain the elasticity “E,” airway resistance “R” and coefficient “a” which can reflect the weight of the step mechanical characteristic. At this time, the SSR value can be obtained by Eq. 38 as the score of the PSO function based on the predicted respiratory volume $\hat{V}$ (t) and the measured volume V(t), and then the velocity vector of N particles is continuously updated for M rounds to find a better value of x (corresponding to the position of the particle), thus obtaining a lower SSR value corresponding to the optimized parameter x. The main flow of the proposed Algorithm 1 is shown in the pseudo-code. The NCP in the pseudo-code stands for non-coefficient parameters. P_SSR_best_, G_SSR_best_, P_best_, G_best_, represent the particle best SSR value, the global best SSR value, the particle best location, and the global best location, respectively.

Algorithm 1 Pseudo-code for models with PSO algorithm.

input:
Data -- Respiratory mechanical data P(t), V’ (t), and V(t)
D -- Number of NCPs
M -- Max number of iterations
N -- Swarm size
LB -- Lower boundary of the search space
UB -- Upper boundary of the search space
output:
G_best_ -- the best positions (NCPs) found so far
(1) Initialization;
(2) **for** each particle i = 1 to N do
(3) **for** each NCP j = 1 to D do
(4) Initialize particle velocity and position randomly within [LB, UB]^D^;
(5) end for
(6) Substituting Data into Eq. 37 to determine all the model parameters;
(7) Compute predicted respiratory volume $\hat{V}$ (t) based on respiratory mechanical model;
(8) Substituting $\hat{V}$ (t) and V(t) to Eq. 38 to initialize P_SSR_best_ to the initial value;
(9) **end for**
(10) Initialize G_best_ and G_SSR_best_ based on the lowest P_SSR_best_;
(11) **while** iteration < max generation M
(12) **for** each particle i = 1 to N do
(13) Update the velocity and position of particle i
(14) **if** the position of particle i exceeds the boundary [LB, UB]^D^ then
(15) the position of particle i is set to the boundary value;
(16) Substituting Data into Eq. 37 to determine all the model parameters;
(17) Compute predicted respiratory volume $\hat{V}$ (t) based on respiratory mechanical model;
(18) Substituting $\hat{V}$ (t) and V(t) to Eq. 38 to calculate temporary SSR (fitness) value;
(19) **if** P_SSR_best_ > temporary SSR value then
(20) P_SSR_best_ = temporary SSR value;
(21) P_best_ = location of particle i
(22) **if** G_SSR_best_ > P_SSR_best_ then
(23) G_SSR_best_ = P_SSR_best_;
(24) G_best_ = P_best_;
(25) **end for**
(26) **end while**

Furthermore, for the Grünwald-Letnikov discretization calculation formula of the open-source vfoderiv3 module in Simulink, you can refer to the following Eqs 41, 42 or the instruction file and related literature of this module (Scherer et al., 2011; Sierociuk et al., 2015; Sierociuk, 2023; Xue and Bai, 2023). When the step size h is sufficiently small and the start time involved in the calculation is t_0_, the calculation of fractional calculus under the Grünwald-Letnikov definition is as follows:

$$D_{t_{0}}^{\alpha }f\left(t\right)=\frac{1}{h^{\alpha }}\sum_{j=0}^{\frac{t-t_{0}}{h}}\omega_{j}f\left(t-jh\right)$$ (41)

where the coefficient $\omega_{j}$ in the above formula (41) has the following recursive formula that is convenient for numerical calculation:

$$\omega_{0}=1,\omega_{j}=\left(1-\frac{\alpha +1}{j}\right)\omega_{j-1},j=1,2,\cdots$$ (42)

For instance, applying it to calculate the α fractional derivative of the respiratory volume V(t) at a certain moment t, is to substitute the measured values of V (t_0_) to V(t) into Eq. 41 for calculation. The coefficients $\omega_{j}$ of Eq. 41 are determined by recursive calculation from Eq. 42.

## 3 Results

### 3.1 Our results indicate that the parameter estimation and model simulation methods proposed in this paper are fast and effective for all the considered classes of models

Four representative different models have been employed to analyze 150 s of PC-AC mode data (corresponding to case1PCAC1.txt, 15,000 data points) for parameter estimation and model simulation. Results for the different models are summarized in Table 1 along with their mean computation time obtained from three runs (all the calculations are retained to four decimal places).

**TABLE 1:** Parameter estimation and simulation results of four representative models.

Model	Parametric estimation	BIC	Correlation	Time (s)
P = EV + RV’ + P0	*p* = 26.1130 V + 7.1716 V’ + 3.3972	**8138.3069**	0.9975	0.2721
P = EV + RV’ + IV'’ + P0	*p* = 26.1135 V + 7.1716 V’ + 0.0085 V'’ + 3.3972	**8427.2498**	0.9975	0.4648
P = EV + RV’ + P0 + a*V^2	*p* = 22.0017 V + 7.1795 V’ + 11.4413 V^2 + 3.4846	**9474.4923**	0.9973	0.2880
P = EV + RV’ + a*D1.1(V) + P0*	*p* = 26.3247 V + 6.6647 V’ + 0.4388D1.1(V) + 3.3804	**1772.4650**	0.9984	0.6916

Note: *D1.1(V) represents the 1.1-order fractional derivative of V.

That the bold values indicates the important BIC indicator results.

**FIGURE 2:**
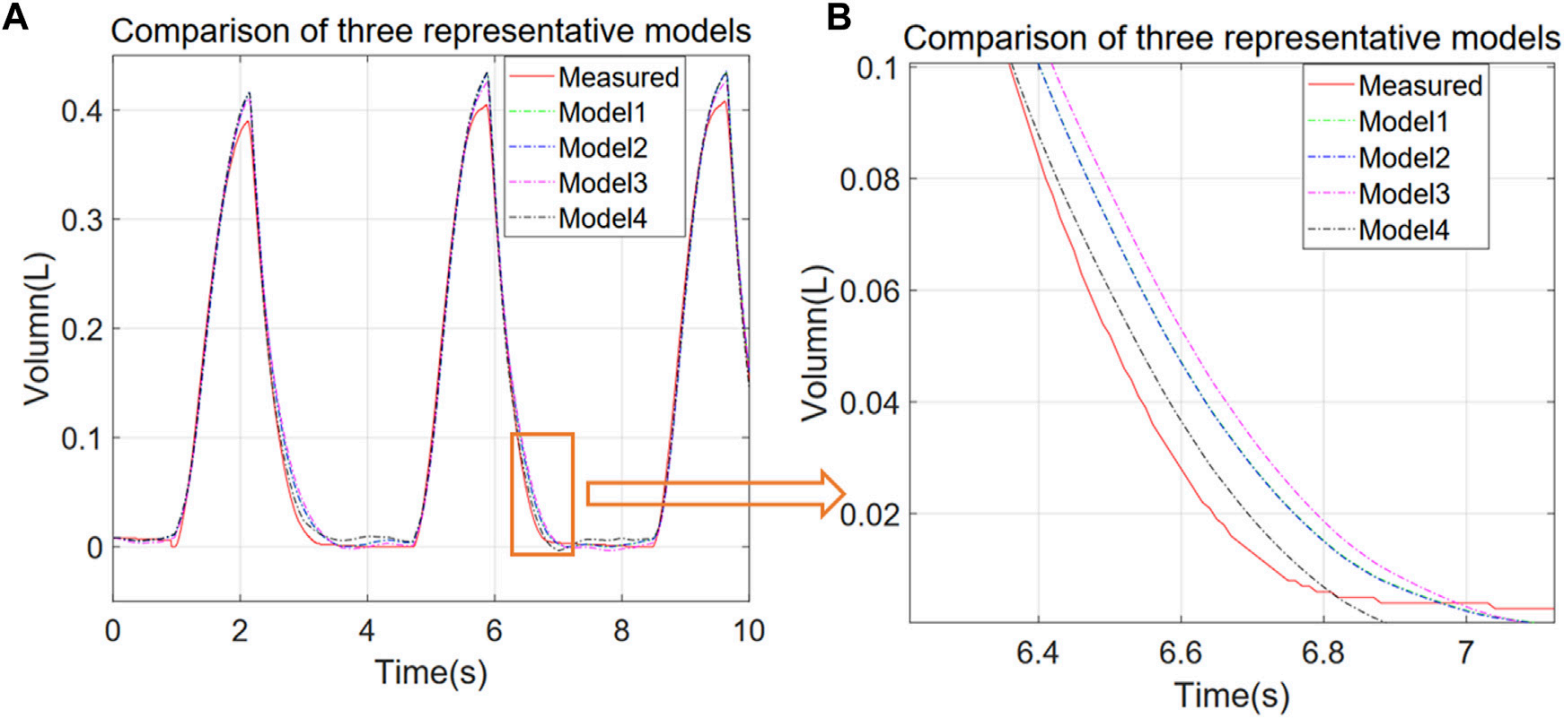
Comparison of experimental and simulated V-T curves for different representative models. Models 1 to 4 correspond to P = EV + RV’ + P0, P = EV + RV’ + IV'’ + P0, P = EV + RV’ + P0 + a*V^2, and P = EV + RV’ + a*D1.1 (V) + P0 respectively. **(A)** 10 s simulation results for each model, **(B)** zoom of the orange region in **(A)**.

As it is apparent from Table 1, the fractional model has the best performance, although the computation time is slightly higher than the other models. For all the models, parameter estimation and model simulation and evaluation may be completed within seconds. Figure 2 shows that models reproduce accurately the measured respiratory volumes V(t) data.

Looking at the first row of Table 1 as an example, we see that the values of respiratory system elastance E = 26.1130 cmH2O/L and respiratory system resistance R = 7.1716 cmH2O*s/L obtained from Eq. 37 both fall within the known range of normal measurements (Protti et al., 2011; Henderson et al., 2017).

In addition, compared to the study by Albanese et al. (2016), our modelling provides an improvement in accuracy by an order of magnitude in terms of the CV(RMSE). In particular, their model achieved an optimal CV(RMSE) = RMSE/average (V(t)) = 0.079 for the volume V(t). While just using the most basic Eq. 1 we find CV(RMSE) = 0.0089 (the results of other models are even better). This demonstrates the accuracy and reliability of our modelling and parameter estimation methods.

### 3.2 The double-exponential model can be chosen as a representative of exponential models

**FIGURE 3:**
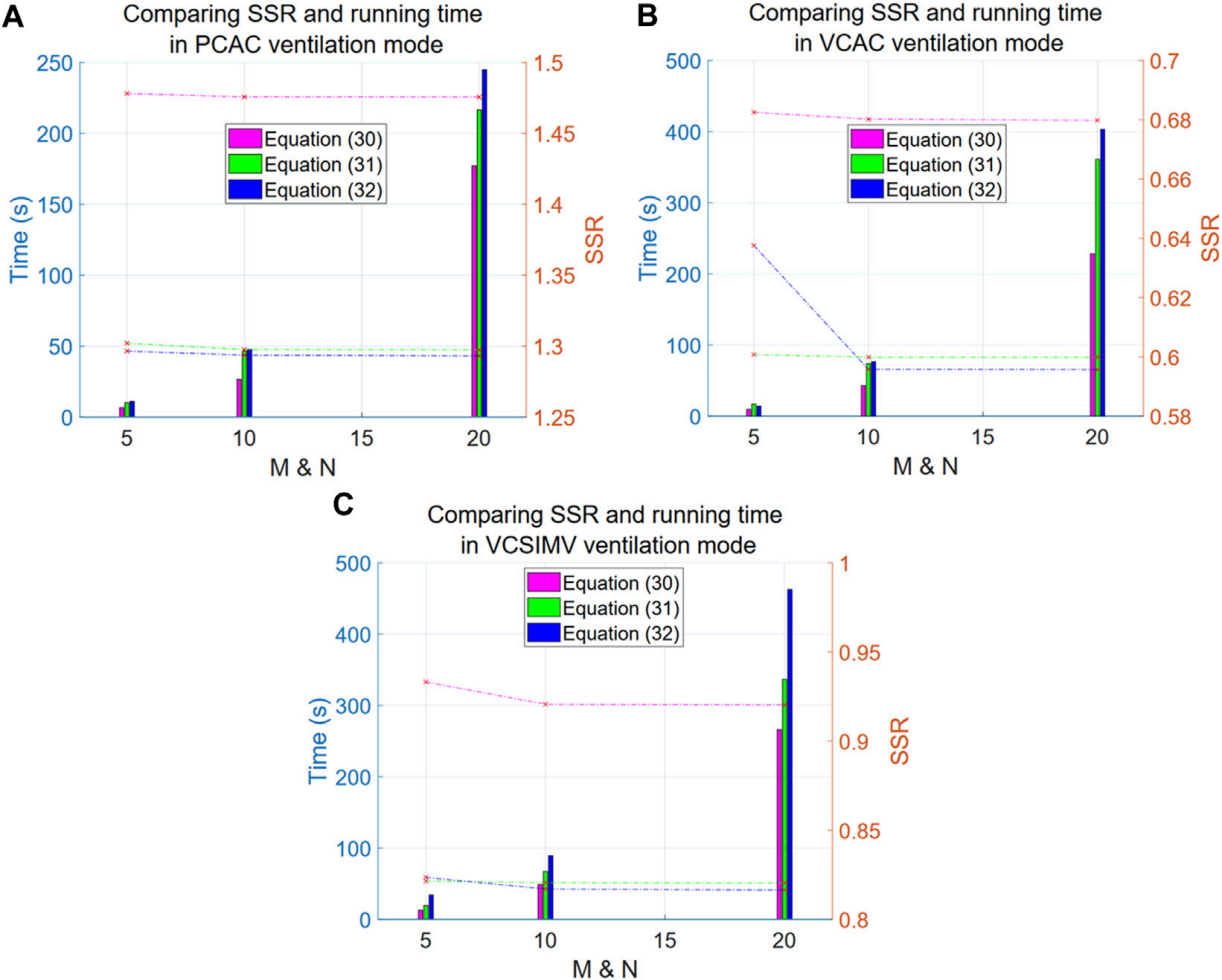
Comparison of SSR and computation time for Eqs 30–32 in three respiratory modes. The left *y*-axis in the Figure denotes time (in seconds), corresponding to the line graph, while the right *y*-axis represents the SSR values, corresponding to the bar graph. The *x*-axis is the particle number N (equal to the number of iterations M). The purple line and bar denote results for Eq. 30, green is for Eq. 31, and blue is for Eq. 32. **(A)** reports results for the PC-AC ventilation mode, **(B)** for the VC-AC ventilation mode and **(C)** for the VC-SIMV ventilation mode.

As mentioned above, we have proposed the exponential Eqs 30–32 to describe data measured in three different ventilation modes: PC-AC, VC-AC, VC-SIMV (corresponding to data files case1PCAC1.txt, case1VCAC1.txt, case1VCSIMV1.txt). The x parameter in the exponentials of Eqs 30–32 is optimized using PSO to obtain the most suitable value, and the criterion for optimization is the SSR value (lower SSR value is better). The number of particles N and the number of iterations M are taken as 5, 10, 20, respectively. The SSR and computation time of each model are summarized as shown in Figure 3.

Overall, the computational results improve (lower SSR values) as the exponent increases (i.e., at a faster growth rate), suggesting that the respiratory system elasticity E has certain step characteristics (the exponential term corresponding to the component separated from the elasticity E), and the closer is to the step characteristics, the better are the results.

Looking at the dashed line in the graph, we conclude that Eq. 32 shows a significant improvement compared to Eq. 30 (with a significant difference of *p*-value = 0.0001009, and an average difference rate of 12.91% in the three ventilation modes). However, there are no significant differences between Eq. 32 and 31 (with a non-significant difference of *p*-value = 0.8271, and an average difference rate of 1.05% in the three ventilation modes). Further increasing the exponential order results only in an increase of the computational time. We thus recommend using the double exponential model as a representative of this type of models. The particle number N and iteration rounds M for this type of model PSO optimization are both set to 10, which allows us to achieve saturation in calculations.

### 3.3 Exponential models provide superior results compared to the polynomial models in volume-controlled ventilation mode

**FIGURE 4:**
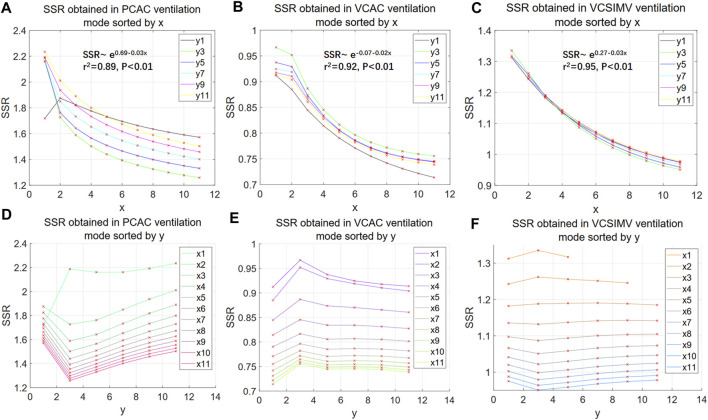
SSR of nonlinear polynomial models. **(A–C)** illustrate the behaviour of the SSR as a function of the exponent x of the aV(t)^x^ term in Eq. 27. The fitting functions corresponding to the mean of all curves in the 3 ventilation modes are shown in Figures 5A–C, respectively (retaining 2 decimal places). **(D–F)** illustrate the behaviour of the SSR as a function of the exponent y of the bV′(t)^y^ term in Eq. 27. The numbers in x1, x2. and y1, y2. in the legend represent the values corresponding to x and y in Eq. 27.

In order to investigate and find out a suitable nonlinear model to describe the actual respiratory data, we simulate the dynamics of Eq. 27, with the exponent ranging from 1 to 11, and select the best model based on its SSR value. When x = 1 Eq. 27 is equivalent to Eq. 29, whereas for y = 1 Eq. 27 is equivalent to Eq. 28. When both x = 1 and y = 1, Eq. 27 reduces to (1). We use three different ventilation modes, PC-AC, VC-AC and VC-SIMV, to estimate the parameters of each model using measured data (corresponding to data files case1PCAC1.txt, case1VCAC1.txt, case1VCSIMV1.txt), and only the parameter values that meet the Routh-Hurwitz criterion and the actual physical meaning are taken for subsequent simulation calculations of SSR values. The results may be summarized as follows:

Figure 4 shows that the exponent y only weakly influences the overall SSR. To reduce the undetermined parameters Eq. 28 is thus taken as the more suitable model for the discussion in the subsequent sections.

The SSR decreases with the exponent x in the term aV(t)^x^ with an approximately exponential behaviour. We take the mean and the logarithm of the mean of the SSR values of all curves in Figures 4A–C respectively, and through linear regression analysis, we find that the logarithm of the mean of the SSR is more significantly correlated to the independent variable x (higher coefficient of determination r2, see Supplementary Material Section 11). This suggests that the SSR of Eq. 28 converges slowly and other may be more convenient in terms of convergence and model fitting.

To simplify and more clearly illustrate our results, we further take the best results obtained by using polynomial Eqs 27–29 of orders 1–11, and compare them with results obtained from Eq. 31. Comparison is made in terms of SSR for the three different ventilation modes, PC-AC, VC-AC, VC-SIMV, for parameter estimation and simulation (corresponding to data files case1PCAC1.txt, case1VCAC1.txt and case1VCSIMV1.txt). For the double exponential Eq. 31, the exponent x is optimized by PSO for a number of particles N = 10 and iterations M = 10.

As can be seen from the results in Table 2, in the Volume-Controlled ventilation mode (VC-AC, VC-SIMV), the double exponential model Eq. 31 has significantly superior BIC and SSR, obtained in a computational time of the same order of magnitude compared with the other models. According to Eq. 26, the exponential term $a{e^{e}}^{xV\left(t\right)}V\left(t\right)$ in Eq. 31 and the aV(t)^x^ term in Eq. 28 are physically related to the elasticity E (component of respiratory elasticity E), and the results of this Section indicates that the elasticity E of the respiratory system has a step-like characteristic, which is more suitable to be modeled and described by exponential or similar fast-growing functions.

**TABLE 2:** Comparison of double-exponential model and polynomial models.

Ventilation modes	Model	BIC	SSR	RSME	Time (s)
PC-AC	best of Eqs 1, 27–29	3513.5670	1.2583	0.0092	39.3937
	Eq. 31	3955.5499	1.2976	0.0093	46.6033
VC-AC	best of Eqs 1, 27–29	−5009.2941	0.7138	0.0069	43.7812
	Eq. 31	**−7615.9289**	**0.5999**	**0.0063**	**74.0008**
VC-SIMV	best of Eqs 1, 27–29	−692.1555	0.95063	0.0080	55.3215
	Eq. 31	**−2916.1684**	**0.8207**	**0.0074**	**127.8307**

That the bold values indicates superior model results in volume-controlled ventilation modes.

### 3.4 The fractional calculus model provides superior results compared to integer-order differential models in Pressure-Controlled ventilation mode

We further compare results for the Pressure-Controlled ventilation mode, introducing the fractional calculus term into the respiratory mechanical model.

Before comparing Eq. 21 with other different models, the undetermined fractional order in the fractional calculus model of Eq. 21 should be estimated using the PSO algorithm to obtain the optimal solution. First, we use PSO algorithm to determine for which values of the particle number N and iteration rounds M one obtains a stable optimal solution for Eq. 21. We use data files from three different breathing patterns: case1PCAC1.txt, case1VCAC1.txt, case1VCSIMV1.txt, and compare SSR for N and M ranging from 3 to 30.

**FIGURE 5:**
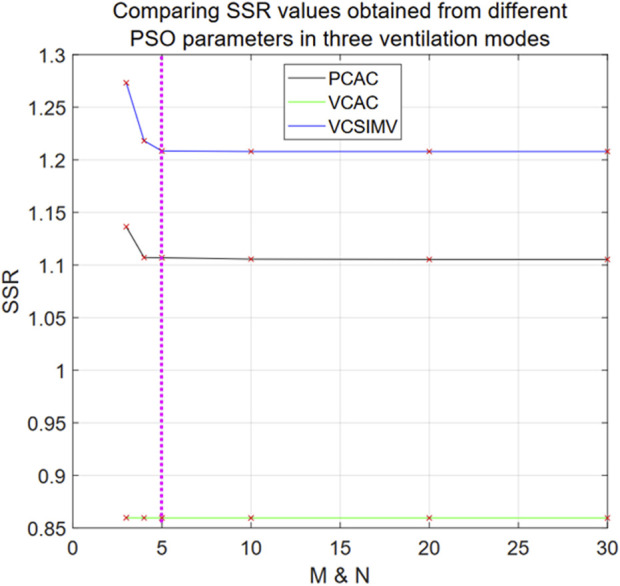
SSR values as obtained from different PSO parameters in the three ventilation modes for Eq. 21. The black line is for the PC-AC mode, green for VC-AC mode, and blue for VC-SIMV mode. The horizontal axis represents the values of M and N in the PSO algorithm, for example, the position of x = 5 denotes N = 5 and M = 5. The SSR value data points calculated from different M and N values are marked with red “x” in the Figure.

The results are shown in Figure 5. Using M = N = 5, PSO already reaches a stable optimal solution, and increasing N and M provides only little improvement on the final result. For N = M = 5, the maximum difference rate from the optimal SSR (N = 30 and M = 30) in the three ventilation modes is 0.14% for the PC-AC mode. Hence, for the PSO algorithm optimization of Eq. 21 we consistently set N = M = 5.

After determining that Eq. 21 has reached a stable optimal solution based on the PSO algorithm with particle number N = 5 and iteration number M = 5, we can further compare the single-compartment model Eq. 1, fractional calculus model Eq. 21, polynomial model Eqs 27–29, and double-exponential model Eq. 31 based on the actual measured data from the three ventilation modes case1PCAC1.txt, case1VCAC1.txt, case1VCSIMV1.txt. We calculate their respective BIC values, SSR values, and computation time. The PSO algorithm is used to optimize the fractional order of Eq. 21 and the exponential parameter of Eq. 31, with the goal of minimizing SSR. In the optimization calculation based on the PSO algorithm, for Eq. 21, the particle number N = 5 and the iteration number M = 5 are taken, while for Eq. 31, according to the previous results, N = M = 10 is taken to achieve saturation. To simplify and clearly present our results, the best results from the polynomial models up to order 11 are compared, and the results are shown in Table 3.

**TABLE 3:** Comparison of fractional calculus model and integer-order differential models.

Ventilation modes	Model	BIC	SSR	RMSE	Time (s)
PC-AC	Eq. 1	8138.3069	1.7171	0.0107	0.2692
	Eq. 21	1571.9530	1.1069	0.0086	24.7259
	best of Eqs 27–29	3513.5670	1.2583	0.0092	39.3937
	Eq. 31	3955.5499	1.2976	0.0093	46.6033
VC-AC	Eq. 1	−1352.4948	0.9120	0.0078	0.2641
	Eq. 21	−2223.0448	0.8595	0.0076	26.7856
	best of Eqs 27–29	−5009.2941	0.7138	0.0069	43.7812
	Eq. 31	−7615.9289	0.5999	0.0063	74.0008
VC-SIMV	Eq. 1	4106.5277	1.7494	0.0108	0.2403
	Eq. 21	2887.3673	1.2084	0.0090	27.9563
	best of Eqs 27–29	−692.1555	0.9506	0.0080	55.3215
	Eq. 31	−2916.1684	0.8207	0.0074	127.8307

From the above results, it is apparent that the fractional calculus model performs better (lower BIC and SSR values) compared to the single-compartment Eq. 1 for all the ventilation modes. The running time is generally within half a minute on our computing platform. The additional computation time of the fractional order model is mostly due to the iterative search, required to find the suitable order, whereas the computational time required for saturation is shorter compared to the exponential Eq. 31. In particular, for the Pressure-Controlled ventilation mode (PC-AC), the fractional calculus model of Eq. 21 is superior to other types of models.

### 3.5 Significant improvement of respiratory model fitting based on hybrid model

As illustrated in the previous Sections, Eq. 21 is optimal for the Pressure-Controlled ventilation mode and Eq. 31 for the Volume-Controlled ventilation mode. In this Section, we investigate whether the hybrid model in Eq. 33, which has features of both models may achieve better results (lower BIC and SSR values) for all the ventilation modes.

Since the fractional order α and the exponential term parameter β in the hybrid Eq. 33 are obtained by PSO algorithm, we first calculate the number of particles N and the number of iterations M required to achieve stable optimal solution based on data from the three different ventilation modes, case1PCAC1.txt, case1VCAC1.txt and case1VCSIMV1.txt.

**FIGURE 6:**
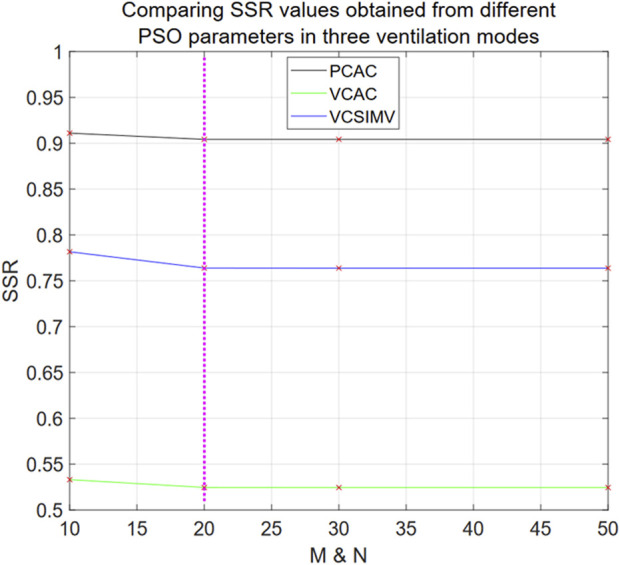
SSR values obtained from different PSO parameters in three ventilation modes for Eq. 33. The horizontal axis denotes the values of M = N in the PSO algorithm, and the SSR value data points calculated from different M and N values are marked with red “x” in the Figure.

From Figure 6, we see that the hybrid Eq. 33 reaches stable optimal solutions for all ventilation modes when N = M = 20, and that there is no statistical difference compared with the optimal SSR (N = 50 and M = 50) (*p*-value = 0.1835, average difference rate 0.0087% in three ventilation modes). In the following, we use N = M = 20 to optimize the parameters of the hybrid model Eq. 33.

**FIGURE 7:**
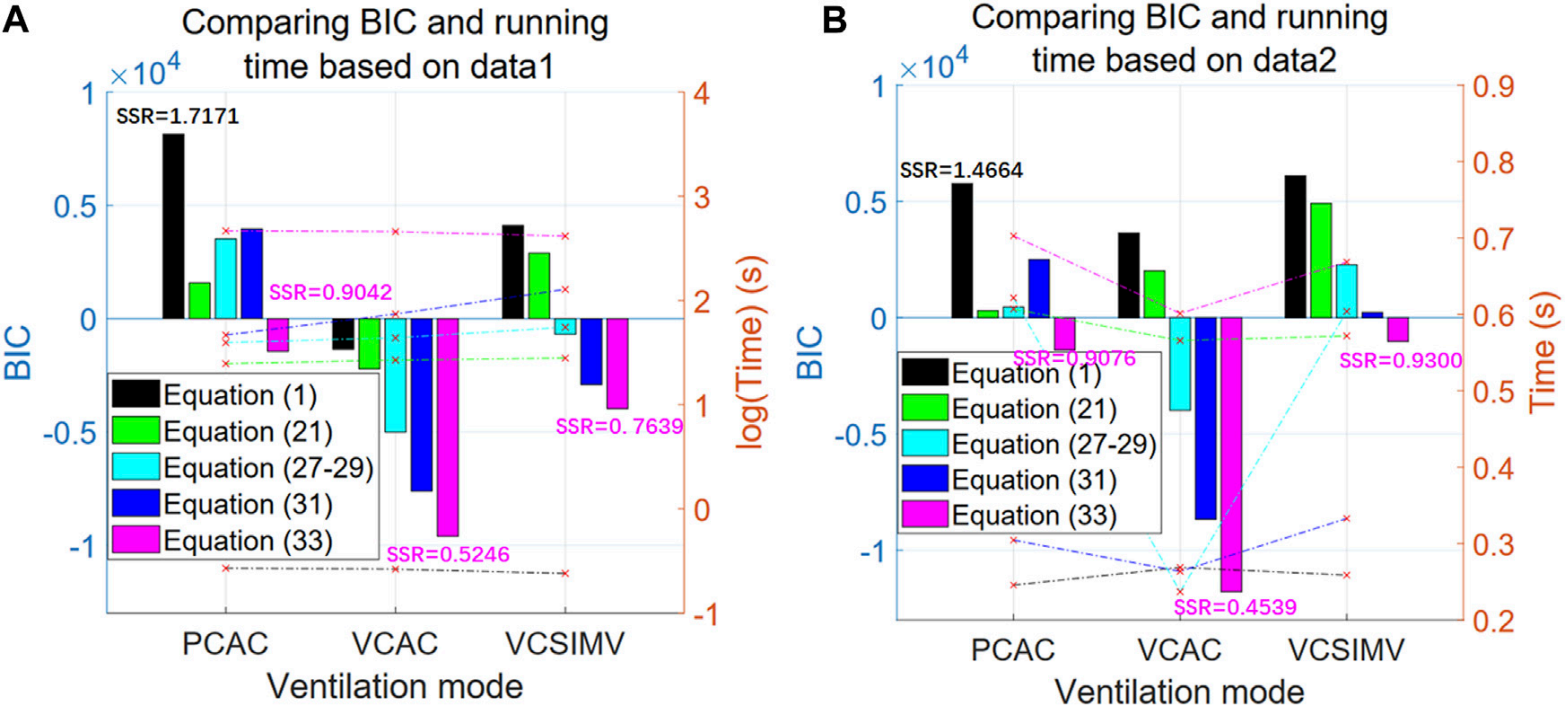
BIC and computational time for the three respiratory modes. The left axis in the figure represents BIC value (bar graph), while the right axis represents time (line graph). The horizontal axis represents three different ventilation modes [black for Eq. 1, green for (Eq. 21), cyan for (Eqs 27–29), blue for (Eq. 31), and purple for (Eq. 33)]. **(A)** The results based on data from the first time period (case1PCAC1.txt, case1VCAC1.txt, case1VCSIMV1.txt). **(B)** The cross-validation results based on data from the second time period (case1PCAC2.txt, case1VCAC2.txt, case1VCSIMV2.txt), with the parameter estimation based on data from the first time period.

Next, we compare the BIC and computational time of the single-compartment model Eq. 1, fractional calculus model Eq. 21, polynomial model Eqs 27–29, double-exponential model Eq. 31 and hybrid model Eq. 33 using the same data (case1PCAC1.txt, case1VCAC1.txt, case1VCSIMV1.txt). For the polynomial model Eqs 27–29, we take the best results from orders 1–11. For Eqs 21, 31, 33, which need to be optimized based on the PSO algorithm, we take their corresponding stable optimal solutions, i.e., N = M = 5, N = M = 10 and N = M = 20, respectively. The results are shown in Figure 7A. After parameter estimation, we further use the relationship between pressure P(t) and respiratory volume V(t) in the data of case1PCAC2.txt, case1VCAC2.txt, case1VCSIMV2.txt for cross-validation (i.e., the pressure in the second time period is used as the input, and the model parameters estimated in the first time period are used for model simulation. The simulated volume $\hat{V}\left(t\right)$ results are compared with the actual measured respiratory volume V(t) in the second time period to calculate SSR and BIC values), and the results of their BIC, SSR values and computational time correspond to Figure 7B.

Results in Figure 7A show that our proposed hybrid model Eq. 33 is the optimal model for all ventilation modes, and that its BIC value is significantly better than other models. When applied to actual measured data from different time periods of the same mode after estimating the model parameters (Figure 7B), similar results are obtained (the behaviour of the BIC for each model is similar), that is, the hybrid model is the optimal model within a given time period and for cross-validation in different time periods.

In terms of computation time, the optimal hybrid model also takes the longest time for parameter estimation stage. The single-compartment model has the worst BIC value and the shortest calculation time (average 0.26 s). Since the hybrid model involves two parameters to be estimated, the main overhead in computation is the optimization of parameters by the PSO algorithm. However, if the personalized characteristic parameters are known, the single-round running time for each model is on the same order of magnitude (Figure 7B), i.e., less than 1 s on our computing platform.

In addition, in order to verify the consistency of the results of each model in different experimental animals, we validated results on measured data from another two Bama pig for the VC-AC mode. First, based on the measured data file case2VCAC1.txt, we estimated the parameters and calculated the BIC and SSR values to evaluate the results of each model. Meanwhile, the model parameters obtained from case2VCAC1.txt were used for cross-validation of the relationship between pressure P(t) and respiratory volume V(t) in the measured data of case2VCAC2.txt. Moreover, the validation for case3 data (case3VCAC1.txt and case3VCAC2.txt) is the same. The calculated results of BIC and SSR values are summarized in Table 4.

**TABLE 4:** Validation of results based on VC-AC mode case2 and case3 data.

Data	Model	BIC	SSR	RMSE	Time (s)
case2VCAC1	Eq. 1	1248.1023	1.0847	0.0085	0.2199
	Eq. 21	160.5779	1.0075	0.0082	18.9196
	best of Eqs 27–29	−4093.9475	0.7587	0.0071	29.5528
	Eq. 31	−6729.8034	0.6364	0.0065	164.6210
	Eq. 33	−8165.1034	0.5776	0.0062	354.8269
case2VCAC2	Eq. 1	43.8811	1.0010	0.0082	0.2289
	Eq. 21	−1070.1428	0.9282	0.0079	0.5648
	best of Eqs 27–29	−4286.4003	0.7490	0.0071	0.2439
	Eq. 31	−6738.7875	0.6361	0.0065	0.3261
	Eq. 33	−8535.8186	0.5635	0.0061	0.6710
case3VCAC1	Eq. 1	6291.3048	1.5182	0.0101	0.2427
	Eq. 21	5104.4553	1.4009	0.0097	20.0965
	best of Eqs 27–29	−531.4728	0.9609	0.0080	30.1726
	Eq. 31	−3296.9257	0.8001	0.0073	100.0014
	Eq. 33	−5174.8426	0.7051	0.0069	365.5986
case3VCAC2	Eq. 1	6846.6370	1.5754	0.0102	0.1550
	Eq. 21	6359.7085	1.5231	0.0101	0.1769
	best of Eqs 27–29	−1166.6333	0.9210	0.0078	0.5751
	Eq. 31	−4718.4334	0.7278	0.0070	0.1951
	Eq. 33	−5374.2667	0.6958	0.0068	0.2108

The validation results are consistent with the results in Figure 7, indicating that the hybrid model Eq. 33 can achieve significantly better results for different ventilation modes. Compared with the basic single-compartment model Eq. 1, the SSR value of hybrid model Eq. 33 is nearly halved and the difference in BIC value is even more significant. In addition, the results of Table 4 also re-verify that if personalized characteristic parameters are obtained based on several respiratory cycle data, they can be used for simulations under the same ventilation mode, and even predict the respiratory volume V(t) curve based on the specified pressure P(t), without having to recalculate the parameter values, with a single round of calculation that can be completed in seconds.

In summary, if there is sufficient computing power or quick calculations is not required, the hybrid model is recommended as the optimal model to universally describe different ventilation modes. When quick calculations are required, the fastest model is the basic Eq. 1. For the same individual, the model parameters that conform to its personalized characteristics can also be pre-calculated based on measured data (Figure 7B), and respiratory volume V(t) can be directly calculated based on pressure P(t) in the same conditions.

## 4 Discussion

We have addressed effective modelling of respiratory mechanics and put forward a set of methods for rapid parameter estimation based on measured data (each model round calculation is less than 1 s), model simulation and evaluation system, also providing the corresponding executable code (https://gitee.com/lizwtest/mechanical_ventilation) and measured data. Using our approach, different models and parameters can be selected and tailored to different applications to further explore the mechanical characteristics of the respiratory system, improve the ventilation mode of the ventilator, and lay the foundation for the implementation of rapid adaptive parameter ventilation.

Some models analyzed in the article have time-domain general solutions, and so when P(t) in the respiratory system is very close to some elementary functions (or multiple superpositions), the volume V(t) curve (or multiple solutions superposition in linear systems) can be obtained directly through the time-domain general solution. However, considering certain autonomous randomness of individual’s breathing and the presence of background, more realistic simulations may be obtained by substituting each point in time order.

In addition, the actual elasticity E of respiratory systems shows a step characteristic. In Volume-Controlled ventilation modes, the pressure P(t) is not fixed, and decreases as it approaches the expected tidal volume, causing a sudden change in the pressure P(t) and respiratory volume V(t) curve. In Pressure-Controlled ventilation modes, the pressure is fixed, which reflects the memory-dependent viscoelasticity and heterogeneity characteristics of lung tissue, whereas in volume-controlled ventilation modes, the pressure P(t) has not a fixed value. The pressure P(t) decreases when it is close to the expected tidal volume, and if it reaches the thoracic/lung volume, the elasticity E of the respiratory system will significantly increase (a larger pressure increment ΔP is required to produce the same volume increment ΔV). This corresponds to a sudden change in the pressure P(t) and volume V(t) curve, and the volume V(t) curve is more likely to reflect the plateau feature. In Pressure-Controlled ventilation modes, the pressure is fixed to reflect the memory-dependent viscoelasticity and heterogeneity characteristics of lung tissue.

Finally, using the systematic approach proposed in this paper, we have proposed and validated an optimal hybrid model useful for various ventilation modes. To this aim, we have for the first time applied fractional calculus and double exponential terms to the modelling of respiratory mechanics. Compared to other models, the hybrid one shows significantly better BIC and SSR results for different ventilation modes, i.e., it better describes the actual pressure P(t) and respiratory volume V(t) relationship. This implies that we can more accurately describe and simulate the characteristics of respiratory mechanics. Based on this model, we can further study the mechanical characteristics of the respiratory system, deepen our understanding of the impact of mechanical ventilation on patients, and optimize the ventilation mode of the ventilator to improve ventilation effects and reduce ventilation damage.

Furthermore, after pre-calculating personalized parameters using several respiratory cycles of different individuals, we can also implement rapid adaptive parameter ventilation based on the hybrid model. Traditional ventilation mode parameters are generally fixed and cannot be dynamically adjusted according to the patient’s real-time respiratory characteristics. However, in the hybrid and other models and methods mentioned in this article, we can dynamically adapt the model and estimate the corresponding model parameters according to the actual data measured at different time intervals of individuals. This makes the overall approach more personalized and adaptable to the actual situation of the patient, thereby achieving better results.

## 5 Conclusion

We have proposed a system for parameter estimation, model simulation, and evaluation based on actual measured data, and after carefully comparing different models, we have put forward an optimal hybrid model valid for various ventilation modes. It may not only help doctors to better understand the mechanical characteristics of the respiratory system, but also improve the ventilation mode of the ventilator, enhance the effect of respiratory therapy, and provide strong support for the realization of rapid adaptive parameter ventilation, which is a relevant feature to improve the safety, effectiveness, and personalization level of mechanical ventilation.

## Data Availability

The original contributions presented in the study are included in the article/Supplementary Material, further inquiries can be directed to the corresponding author/s.
