# Supplementary material for: An enhanced respiratory mechanics model based on double-exponential and fractional calculus
Source: Front Physiol. 2023 Dec 4;14:1273645. doi: 10.3389/fphys.2023.1273645 (PMC10726035; doi:10.3389/fphys.2023.1273645)
Supplement: Supplementary file 1 [file DataSheet3.DOCX]

Supplementary Material

# 1. Examples of some general solutions for first order linear differential models

For the main text equation (1) (linear single-compartment model), which takes the form of $y^{'}+P\left( x \right)y=Q(x)$. This form has a general solution $y=Ke^{-\int P(x)dx}+e^{-\int P(x)dx}\int Q(x)e^{\int P(x)dx}dx$, where K is a constant to be determined by the initial conditions.

## 1.1 In the case of airway pressure P being an ideal square wave

When the airway pressure P is an ideal rectangular wave, corresponding P(t) is:

$P(t)=\left\{ \begin{aligned} P_{max} 0\leq t\leq T_{I} \\ 0 T_{I}\leq t\leq T_{T} \end{aligned} \right.$ (1.1.1)

where T_I_ is the inspiratory time, and T_T_ is the total cycle time.

During the inspiration period ($0\leq t\leq T_{I}$), $P(t)=\frac{V(t)}{C}+R\frac{dV(t)}{dt}+P_{0}$ can be transformed into the form of $V’(t)+\frac{V(t)}{\mathrm{CR}}=\frac{P(t)-P_{0}}{R}$ , where C represents the compliance of the respiratory system, and E represents the elasticity of the respiratory system. The general solution corresponding to the undetermined initial conditions is:

$V\left( t \right)=Ke^{-\frac{t}{CR}}+C(P_{max}-P_{0})$ (1.1.2)

The constant K in the above formula is determined by the initial conditions. When P_0_=0 and V(0)=0, it can be obtained that K=-CP_max_. Substituting into equation (1.1.2) yields the result of the following formula:

$V\left( t \right)=CP_{max}\left( 1-e^{-\frac{t}{CR}} \right)$ (1.1.3)

In the expiratory period ($T_{I}\leq t\leq T_{T}$) when the air pressure P is 0, the change in the respiratory volume V(t) can be regarded as a step response from a non-zero initial state, then we have:

$V(t)=\left\{ \begin{aligned} CP_{max}\left( 1-e^{-\frac{t}{CR}} \right) 0\leq t\leq T_{I} \\ CP_{max}(1-e^{-\frac{T_{I}}{CR}})e^{-\frac{t-T_{I}}{CR}} T_{I}\leq t\leq T_{T} \end{aligned} \right.$ (1.1.4)

Furthermore, by taking the derivative of equation (1.1.4), we can obtain the ventilation rate $V'\left( t \right)$.

$V'\left( t \right)=\frac{dV(t)}{dt}=\left\{ \begin{aligned} \frac{P_{max}}{R}e^{-\frac{t}{CR}} 0\leq t\leq T_{I} \\ -\frac{P_{max}}{R}(1-e^{-\frac{T_{I}}{CR}})e^{-\frac{t-T_{I}}{CR}} T_{I}\leq t\leq T_{T} \end{aligned} \right.$ (1.1.5)

Here, we simulate based on the empirical values^[1]^ of the human respiratory system, taking C=0.1L/cmH2O, R=4.3cmH2O*s/L, P_max_=10cmH2O and T_I_=0.5T_T_=3s, which results in the following simulation results:


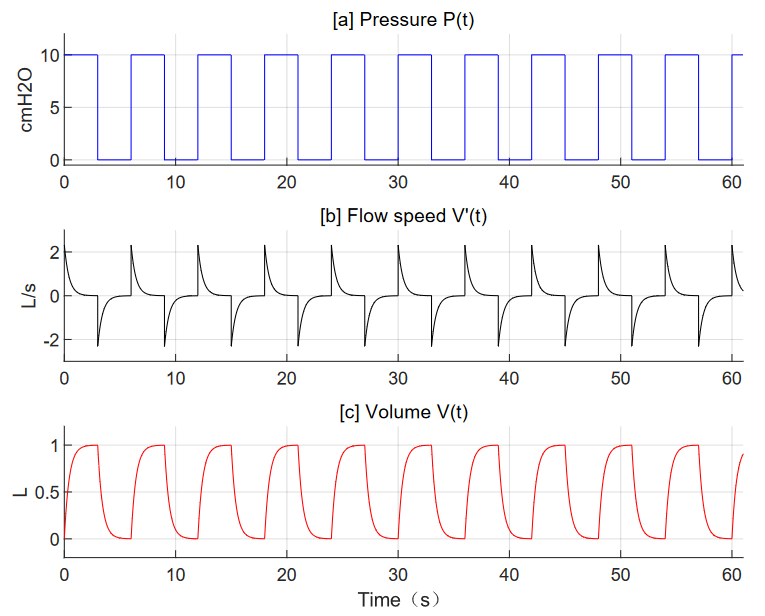


Supplementary Figure 1.1. The corresponding simulation result graph when the airway pressure P(t) is a square wave.

Here, we approximately consider 1 mbar to be approximately equal to 1 cmH2O.

## 1.2 In the case of airway pressure P being a sine wave

Similar to section 1.1, in the case where the airway pressure P(t) is a sine wave, the following equation exists:

$P\left( t \right)=P_{max}sin(at)$ (1.2.1)

At this point, we can obtain $V’(t)+\frac{V(t)}{\mathrm{CR}}=\frac{P_{max}sin(at)-P_{0}}{R}$, and its general solution is:

$V\left( t \right)=Ke^{-\frac{t}{CR}}+\frac{P_{max}C}{a^{2}C^{2}R^{2}+1}\left( sin\left( at \right)-aCRcos\left( at \right) \right)-CP_{0}$ (1.2.2)

The constant K in the above formula is determined by the initial conditions. When P_0_=0 and V(0)=0, it can be obtained that $K=\frac{P_{max}aC^{2}R}{a^{2}C^{2}R^{2}+1}$. Substituting into equation (1.2.2) yields the result of the following formula:

$V(t)=\frac{P_{max}C}{a^{2}C^{2}R^{2}+1}(sin\left( at \right)-aCRcos\left( at \right)+aCRe^{-\frac{t}{CR}})$ (1.2.3)

When the initial conditions are the same, this result is consistent with the result obtained by convolving equation (3) with the sine function based on equation (6) in the main text.

For the actual situation of mechanical ventilation, generally only positive pressure ventilation is carried out, which can be for the situation of formula (1.2.4):

$P(t)=\left\{ \begin{aligned} P_{max}sin(at) 0\leq t\leq\pi\\ 0 \pi\leq t\leq T_{T} \end{aligned} \right.$ (1.2.4)

In the expiratory period ($\pi\leq t\leq T_{T}$) when the air pressure P is 0, the change in the respiratory volume V(t) can be regarded as a step response from a non-zero initial state, then we have:

$V\left( t \right)=\left\{ \begin{aligned} \frac{P_{max}}{{a^{2}C}^{2}R^{2}+1}(Csin\left( at \right)-aC^{2}Rcos\left( at \right)+aC^{2}Re^{-\frac{t}{CR}}) 0\leq t\leq\pi\\ \frac{P_{max}}{{a^{2}C}^{2}R^{2}+1}(aC^{2}R+aC^{2}Re^{-\frac{\pi}{CR}})e^{-\frac{t-\pi}{CR}} \pi\leq t\leq T_{T} \end{aligned} \right.$ (1.2.5)

The derivative based on (1.2.5) yields the ventilation rate V'(t) as follows:

$V'\left( t \right)=\left\{ \begin{aligned} \frac{P_{max}}{{a^{2}C}^{2}R^{2}+1}(aCcos\left( at \right)+a^{2}C^{2}Rsin\left( at \right)-aCe^{-\frac{t}{CR}}) 0\leq t\leq\pi\\ -\frac{P_{max}}{{a^{2}C}^{2}R^{2}+1}(aC+aCe^{-\frac{\pi}{CR}})e^{-\frac{t-\pi}{CR}} \pi\leq t\leq T_{T} \end{aligned} \right.$ (1.2.6)

Based on equations (1.2.4), (1.2.5) and (1.2.6), taking P_max_=5cmH2O, a=1, C=0.1 L/cmH2O, R=4.3 cmH2O*s/L and $T_{T}=2\pi$, we can obtain the following simulation results:


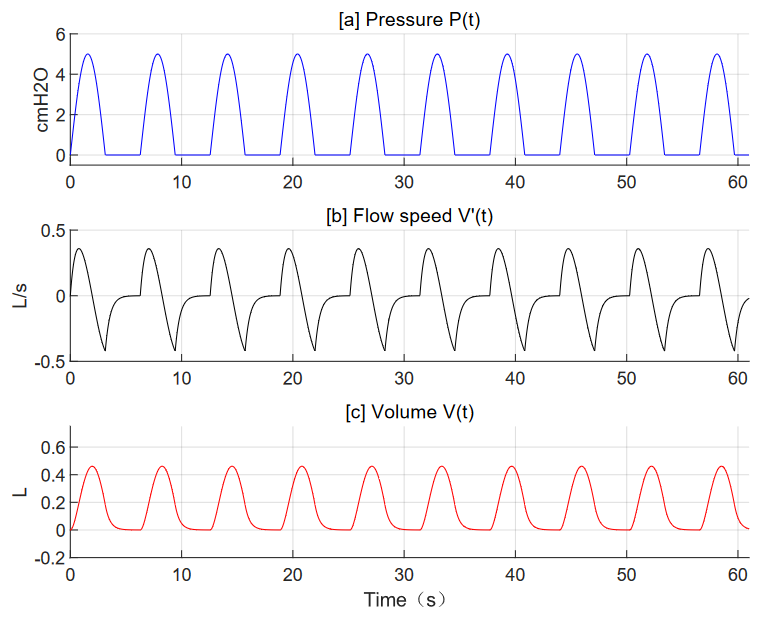


Supplementary Figure 1.2. The corresponding simulation result graph when the airway pressure P(t) is a Sine wave (only the positive half-cycle).

## 1.3 In the case of airway pressure P being an ideal exponential function

When the airway pressure P is an exponential function, corresponding P(t) can have the following form:

$P(t)=\left\{ \begin{aligned} P_{max}(1-e^{-\frac{t}{CR}}) 0\leq t\leq T_{I} \\ P_{max}(1-e^{-\frac{T_{I}}{CR}})e^{-\frac{t-T_{I}}{CR}} T_{I}\leq t\leq T_{T} \end{aligned} \right.$ (1.3.1)

During the inspiration period ($0\leq t\leq T_{I}$), we can obtain $V’(t)+\frac{V(t)}{\mathrm{CR}}=\frac{P_{max}(1-e^{-\frac{t}{CR}})-P_{0}}{R}$, and its general solution is:

$V\left( t \right)=Ke^{-\frac{t}{CR}}+C\left( P_{max}-P_{0} \right)-\frac{P_{max}}{R}te^{-\frac{t}{CR}}$ (1.3.2)

The constant K in the above formula is determined by the initial conditions. When P_0_=0 and V(0)=0, it can be obtained that K=-CP_max_. Substituting into equation (1.3.2) yields the result of the following formula:

$V\left( t \right)=P_{max}C\left( 1-e^{-\frac{t}{CR}}-\frac{t}{CR}e^{-\frac{t}{CR}} \right)$ (1.3.3)

In the expiratory period ($T_{I}\leq t\leq T_{T}$) when the air pressure P is 0, the change in the respiratory volume V(t) can be regarded as a step response from a non-zero initial state, then we have:

$V(t)=\left\{ \begin{aligned} P_{max}C\left( 1-e^{-\frac{t}{CR}}-\frac{t}{CR}e^{-\frac{t}{CR}} \right) 0\leq t\leq T_{I} \\ P_{max}C{(1-e^{-\frac{T_{I}}{CR}}-\frac{T_{I}}{CR}e^{-\frac{T_{I}}{CR}})(e}^{-\frac{t-T_{I}}{CR}}+\frac{t-T_{I}}{CR}e^{-\frac{t-T_{I}}{CR}}) T_{I}\leq t\leq T_{T} \end{aligned} \right.$ (1.3.4)

When the initial conditions are the same, this result is consistent with the result obtained by convolving equation (3) with equation (1.3.1) based on equation (6) in the main text.

The derivative based on (1.3.4) yields the ventilation rate V'(t) as follows:

$V'\left( t \right)=\left\{ \begin{aligned} \frac{P_{max}t}{CR^{2}}e^{-\frac{t}{CR}} 0\leq t\leq T_{I} \\ \frac{P_{max}(T_{I}-t)}{CR^{2}}(1-e^{-\frac{T_{I}}{CR}}-\frac{T_{I}}{CR}e^{-\frac{T_{I}}{CR}})e^{-\frac{t-T_{I}}{CR}} T_{I}\leq t\leq T_{T} \end{aligned} \right.$ (1.3.5)

Taking P_max_=5 cmH_2_O,C=0.1 L/cmH_2_O,R=4.3 cmH_2_O*s/L and T_I_=0.5T_T_=3s, we can obtain the simulated results of pressure P(t) and respiratory volume V(t) shown in the following graph:


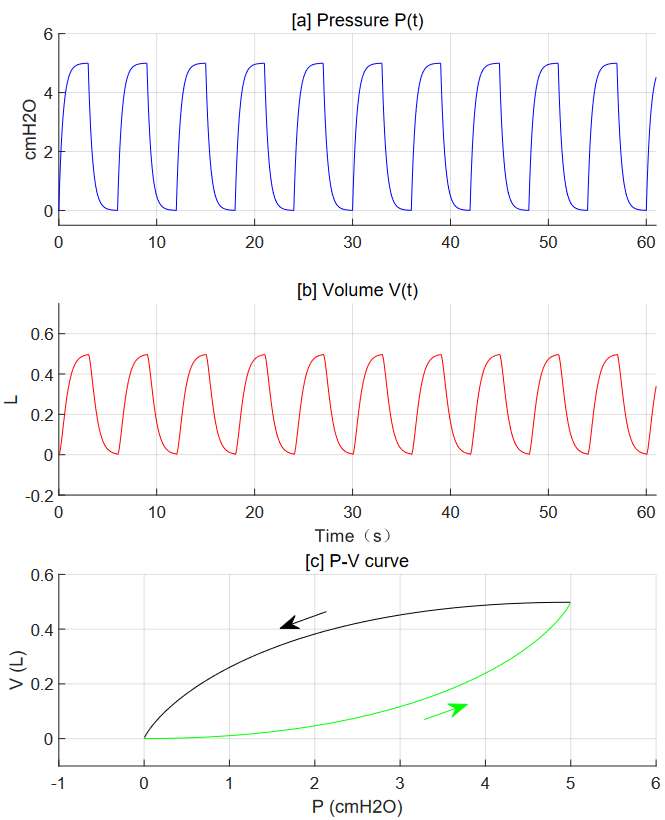


Supplementary Figure 1.3. The corresponding simulation result graph when the airway pressure P(t) is an ideal exponential function. (a) represents pressure P(t), (b) represents respiratory volume V(t), and (c) represents the P-V curve. During the inspiration period ($0\leq t\leq T_{I}$) corresponds to the green line, and the expiratory period ($T_{I}\leq t\leq T_{T}$) corresponds to the black line.

From the simulation results, it can be seen that the exponential input is very similar to the rectangular wave input, with the difference being that the pressure P(t) input and respiratory volume V(t) changes are more gradual.

# 2 Examples of some general solutions for second order linear differential models

For the second-order linear differential models of equation (10) and equation (14), they can be written in the form of a constant coefficient second-order differential equation $y^{''}+py^{'}+qy=f\left( x \right)$, which has a general solution y=K_1_y_1_+K_2_y_2_+y^*^, where K_1_y_1_+K_2_y_2_ is the general solution of the homogeneous equation $y^{''}+py^{'}+qy=0$. The constants K_1_ and K_2_ are determined by the initial conditions and $y^{*}=y_{1}\int\frac{f(x)}{{(\frac{y_{1}}{y_{2}})}^{'}y_{2}}dx+y_{2}\int\frac{f(x)}{{(\frac{y_{2}}{y_{1}})}^{'}y_{1}}dx$. In fact, there are corresponding analytical general solutions for linear differential equations of order less than 5.

## 2.1 In the case of airway pressure P being an ideal square wave

When the airway pressure P(t) is the same square wave as equation (1.1.1), the second-order differential model can be written as follows during the inspiration period ($0\leq t\leq T_{I}$):

$\frac{d^{2}V\left( t \right)}{dt^{2}}+\frac{R}{I}\frac{dV\left( t \right)}{dt}+\frac{1}{IC}V(t)=\frac{P_{max}-P_{0}}{I}$ (2.1.1)

In the actual lung environment, parameter I is usually very small. Its corresponding quadratic equation has two distinct real eigenvalues (for the overdamped situation with no overshoot, the eigenvalues only have negative real parts, then R2>4I/C), and its general solution is:

$V\left( t \right)=K_{1}e^{r_{1}t}+K_{2}e^{r_{2}t}+C(P_{max}-P_{0})$ (2.1.2)

where $r_{1}=\frac{-\frac{R}{I}+\sqrt{\frac{R^{2}}{I^{2}}-\frac{4}{\mathrm{IC}}}}{2}$, $r_{2}=\frac{-\frac{R}{I}-\sqrt{\frac{R^{2}}{I^{2}}-\frac{4}{\mathrm{IC}}}}{2}$ are two distinct real roots corresponding to the characteristic equation (2.1.1), and the constants K_1_ and K_2_ are determined by the initial conditions. When the initial conditions are V(0)=0,$V'\left( t \right)=0$ and P_0_=0, $\left\{ \begin{aligned} K_{1}=\frac{r_{2}}{r_{1}-r_{2}}CP_{max} \\ K_{2}=\frac{r_{1}}{r_{2}-r_{1}}CP_{max} \end{aligned} \right.$can be determined by the constraint $\left\{ \begin{aligned} K_{1}+K_{2}=-CP_{max} \\ K_{1}r_{1}+K_{2}r_{2}=0 \end{aligned} \right.$, that is:

$V\left( t \right)=CP_{max}({\frac{r_{2}}{r_{1}-r_{2}}e}^{r_{1}t}+\frac{r_{1}}{r_{2}-r_{1}}e^{r_{2}t}+1)$ (2.1.3)

In the expiratory period ($T_{I}\leq t\leq T_{T}$)when the air pressure P is 0, the change in the respiratory volume V(t) can be regarded as a step response from a non-zero initial state. The general solution for the expiratory period can also be obtained in the same way as in section 1.1.

The derivative based on (2.1.3) yields the ventilation rate V'(t) as follows:

$V'\left( t \right)=CP_{max}({\frac{r_{1}r_{2}}{r_{1}-r_{2}}(e}^{r_{1}t}-e^{r_{2}t}))$ (2.1.4)

## 2.2 In the case of airway pressure P being a sine wave

If airway pressure P(t) is the sine wave condition similar to (1.2.1), during the inspiration period ($0\leq t\leq T_{I}$), equation (10) can be written in the following form:

$\frac{d^{2}V\left( t \right)}{dt^{2}}+\frac{R}{I}\frac{dV\left( t \right)}{dt}+\frac{1}{IC}V(t)=\frac{P_{max}sin(at)-P_{0}}{I}$ (2.2.1)

The general solution is：

$V\left( t \right)=K_{1}e^{r_{1}t}+K_{2}e^{r_{2}t}-CP_{0}-\frac{aCR\cos\left( \mathrm{at} \right)+(a^{2}CI-1)sin(at)}{a^{2}C^{2}R^{2}+{(a^{2}CI-1)}^{2}}CP_{max}$ (2.2.2)

where $r_{1}=\frac{-\frac{R}{I}+\sqrt{\frac{R^{2}}{I^{2}}-\frac{4}{\mathrm{IC}}}}{2}$, $r_{2}=\frac{-\frac{R}{I}-\sqrt{\frac{R^{2}}{I^{2}}-\frac{4}{\mathrm{IC}}}}{2}$ are two distinct real roots corresponding to the characteristic equation (2.2.1), and the constants K_1_ and K_2_ are determined by the initial conditions.

For equation (14), the general solution corresponding to airway pressure P(t) as a sine wave in equation (1.2.1) is:

$V\left( t \right)=K_{1}e^{r_{1}t}+K_{2}e^{r_{2}t}-\frac{P_{0}}{E}-\frac{a(R+(-a^{2}I+E)k)\cos(at)+(-E+a^{2}(I+\mathrm{Rk}))\sin(at)}{a^{2}R^{2}+{(a^{2}I-E)}^{2}}P_{max}$ (2.2.3)

In the above equation, k is the constant coefficient of P'(t) in equation (14), and the rest are the same as those in equation (2.2.2).

In the expiratory period ($T_{I}\leq t\leq T_{T}$) when the air pressure P is 0, the change in the respiratory volume V(t) can be regarded as a step response from a non-zero initial state. The general solution for the expiratory period can also be obtained in the same way as in section 1.2.

## 2.3 In the case of airway pressure P being an ideal exponential function

When the airway pressure P is an exponential function, corresponding P(t) can have the following form:

${P\left( t \right)=P}_{max}(1-e^{-\frac{t}{\tau}})$ (2.3.1)

In the above equation, $\tau$ represents the time constant (which does not necessarily equal the product of C and R), thus equation (10) can be rewritten as follows:

$\frac{d^{2}V\left( t \right)}{dt^{2}}+\frac{R}{I}\frac{dV\left( t \right)}{dt}+\frac{1}{IC}V(t)=\frac{P_{max}(1-e^{-\frac{t}{\tau}}) -P_{0}}{I}$ (2.3.2)

The general solution is：

$V\left( t \right)=K_{1}e^{r_{1}t}+K_{2}e^{r_{2}t}+C\left( P_{max}-P_{0} \right)-{\frac{C\tau^{2}}{IC+\tau^{2}-CR\tau}e}^{-\frac{t}{\tau}}P_{max}$ (2.3.3)

where $r_{1}=\frac{-\frac{R}{I}+\sqrt{\frac{R^{2}}{I^{2}}-\frac{4}{\mathrm{IC}}}}{2}$, $r_{2}=\frac{-\frac{R}{I}-\sqrt{\frac{R^{2}}{I^{2}}-\frac{4}{\mathrm{IC}}}}{2}$ are two distinct real roots corresponding to the characteristic equation (2.3.2), and the constants K_1_ and K_2_ are determined by the initial conditions.

Similarly, the general solution for equation (14) can be obtained as follows:

$V\left( t \right)=K_{1}e^{r_{1}t}+K_{2}e^{r_{2}t}+\frac{1}{E}(P_{max}-P_{0})-\frac{\tau(k+\tau)ⅇ^{-t/\tau}}{I+\tau\left( -R+E\tau\right)}P_{max}$ (2.3.4)

In the above equation, k is the constant coefficient of P'(t) in equation (14), and the rest are the same as those in equation (2.3.3).

# 3. High-order differential models often lead to system instability

Taking case1PCAC1.txt as an example, if we assume that the respiratory system has the following 3rd or 4th order differential form:

$P(t)=J\frac{d^{3}V\left( t \right)}{dt^{3}}+I\frac{d^{2}V\left( t \right)}{dt^{2}}+R\frac{dV\left( t \right)}{dt}+EV(t)+P_{0}$ (3.1)

$P(t)=S\frac{d^{4}V\left( t \right)}{dt^{4}}+J\frac{d^{3}V\left( t \right)}{dt^{3}}+I\frac{d^{2}V\left( t \right)}{dt^{2}}+R\frac{dV\left( t \right)}{dt}+EV(t)+P_{0}$ (3.2)

The results obtained from the parameter estimation method in equation (27) of the main text are as shown in the following table:

Table S3.1 Results of parameter estimation for equation (3.1) and equation (3.2)

| **Model** | **Parameters** |
| --- | --- |
| equation (3.1) | E=26.1179; R=7.1340; I=0.0245; J=-12.7900; P0=3.3972; |
| equation (3.2) | E=26.1975; R=7.1318; I=1.5891; J=-12.8034; S=10.7411; P0=3.3884; |

From the results of the instance, it can be seen that the parameter estimation of the high-order differential model often results in negative coefficients for high-order terms. Based on Routh-Hurwitz stability criterion, the system will be unstable. For instance, if we conduct further simulations, we obtain the divergent results shown in the following supplementary figure 3, which are consistent with the conclusions in Supplementary Material section 4.


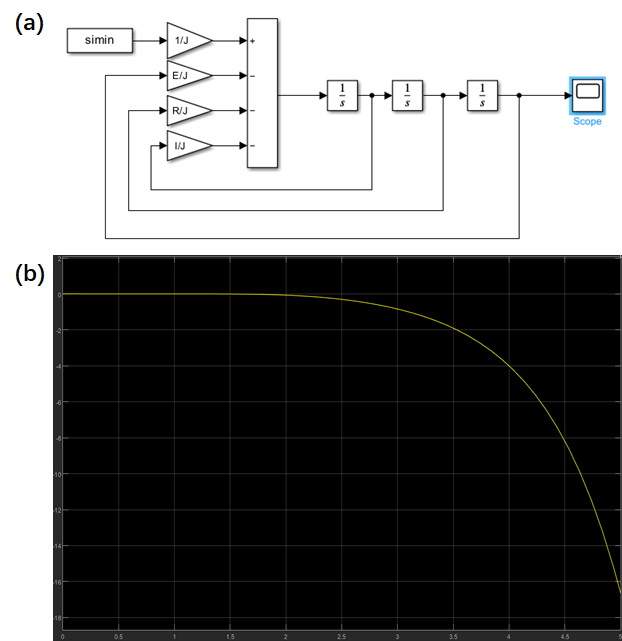


Supplementary Figure 3. (a) represents the Simulink model graph corresponding to equation (3.1) (b) The horizontal axis represents time (s) and the vertical axis represents the respiratory volume (L).

# 4. Discussion on the existence of high-order differential terms in nondegenerate mechanics system

In nondegenerate mechanics system, based the Hamilton's Principle and Ostrogradsky's theorem, terms of order three and higher in higher-order differential models do not exist.

Assume that there are third-order or higher derivative terms in the respiratory system, in the following form:

$P\left( t \right)-P_{0}=a_{0}V(t)+a_{1}\frac{dV\left( t \right)}{dt}+a_{2}\frac{d^{2}V\left( t \right)}{dt^{2}}+\ldots+a_{n}\frac{d^{n}V\left( t \right)}{dt^{n}}$ (4.1)

In the above equation, n>2, integrating both sides of the equation with respect to V(t) yields:

$E=\int(P\left( t \right)-P_{0})dV(t)=\int(a_{0}V(t)+a_{1}\frac{dV\left( t \right)}{dt}+a_{2}\frac{d^{2}V\left( t \right)}{dt^{2}}+\ldots+a_{n}\frac{d^{n}V\left( t \right)}{dt^{n}})dV(t)$ (4.2)

Then we have:

$\left\{ \begin{matrix} E_{p}=\int a_{0}V\left( t \right)dV\left( t \right)=\frac{1}{2}a_{0}{(V(t))}^{2} \\ E_{d}=\int a_{1}\frac{dV\left( t \right)}{dt}dV\left( t \right)=a_{1}\int{(\frac{dV\left( t \right)}{dt})}^{2}dt \\ \begin{matrix} E_{k}=\int a_{2}\frac{d^{2}V\left( t \right)}{dt^{2}}dV\left( t \right)={\frac{1}{2}a}_{2}{(\frac{dV\left( t \right)}{dt})}^{2} \\ \vdots\\ E_{n}=\int a_{n}\frac{d^{n}V\left( t \right)}{dt^{n}}dV\left( t \right)=a_{n}\int\frac{d^{n}V\left( t \right)}{dt^{n}}\frac{dV\left( t \right)}{dt}dt \end{matrix} \end{matrix} \right.$ (4.3)

where E_p_ is potential energy, E_d_ is damping energy dissipation, E_k_ is kinetic energy and the other energy terms are derived from the third order or higher order differential terms.

When the highest order of differential term in the mechanical system model is second order, the system only involves E_p_, E_d_ and E_k_. At this point, the highest order of derivative in the Lagrangian is only 1, that is, $\mathcal{L=L(}V,V',t)$. For simplicity, we can temporarily ignore the energy dissipation due to damping, assuming E_d_=0. Then we have:

$\mathcal{L=}E_{k}-E_{p}={\frac{1}{2}a}_{2}{(\frac{dV\left( t \right)}{dt})}^{2}-\frac{1}{2}a_{0}\left( V\left( t \right) \right)^{2}$ (4.4)

The change in respiratory volume V is equivalent to bringing elastic deformation/displacement to the respiratory system tissues, and when the system has an extreme value, it satisfies the Euler-Lagrange Equation:

$\frac{\partial\mathcal{L}}{\partial V}=\frac{d}{dt}\frac{\partial\mathcal{L}}{\partial V'}$ (4.5)

where $\frac{\partial\mathcal{L}}{\partial V}$ can be regarded as generalized force, and $\frac{\partial\mathcal{L}}{\partial V'}$ can be regarded as generalized momentum. The Hamiltonian H corresponding to this system is the Legendre transformation of the Lagrangian.

$H=\frac{\partial\mathcal{L}}{\partial V'}V^{'}\mathcal{-L=}a_{2}V^{'}\cdot V^{'}-\left( {\frac{1}{2}a}_{2}{V^{'}}^{2}-{\frac{1}{2}a}_{0}V^{2} \right)={\frac{1}{2}a}_{2}{V^{'}}^{2}+{\frac{1}{2}a}_{0}V^{2}$ (4.6)

In the above formula, both $V^{'}$ and V are square terms, and the Hamiltonian H has a lower bound of 0. That is, without considering E_d_ in nondegenerate mechanics system, for model (4.1) when n≤2, the Lagrangian does not contain second-order and higher derivative terms, at this time the Hamiltonian has a lower bound, hence the system is stable.

If there exist third-order or higher differential terms in the mechanical system, then the Lagrangian contains second-order and higher differential terms. (for instance $a_{3}\int\frac{d^{3}V\left( t \right)}{dt^{3}}\frac{dV\left( t \right)}{dt}dt$). Based on Ostrogradsky's theorem, the Hamiltonian H will be unbounded in nondegenerate mechanics system. According to Hamilton's Principle, this will lead to the system evolving in the direction of $H\to-\infty$, resulting in Ostrogradsky instability. Therefore, it is generally not recommended to use models with third-order or higher derivative terms in the respiratory mechanics system.

# 5. Numerical calculation of fractional calculus calculus

For numerical calculations, if the step size h is sufficiently small, the following equation holds:

$\mathcal{D}_{t_{0}}^{\alpha}f(t)=\frac{1}{h^{\alpha}}\sum_{j=0}^{\frac{t-t_{0}}{h}} \omega_{j}f(t-jh)$ (5.1)

and the coefficient $\omega_{j}$ in the above formula (21) has the following recursive formula that is convenient for numerical calculations:

$\omega_{0}=1, \omega_{j}=\left( 1-\frac{\alpha+1}{j} \right)\omega_{j-1} , j=1, 2, \cdots$ (5.2)

According to the method of integration by parts in calculus, the Gamma function has the property$\Gamma\left( z+1 \right)=z\Gamma\left( z \right)$,then we have $\frac{\omega_{j}}{\omega_{j-1}}=\frac{\frac{\left( -1 \right)^{j}\Gamma\left( \alpha+1 \right)}{\Gamma\left( j+1 \right)\Gamma\left( \alpha-j+1 \right)}}{\frac{\left( -1 \right)^{j-1}\Gamma\left( \alpha+1 \right)}{\Gamma\left( j \right)\Gamma\left( \alpha-j+2 \right)}}=-\frac{\Gamma\left( j \right)\Gamma\left( \alpha-j+2 \right)}{\Gamma\left( j+1 \right)\Gamma\left( \alpha-j+1 \right)}=-\frac{\Gamma\left( j \right)\left( \alpha-j+1 \right)\Gamma\left( \alpha-j+1 \right)}{j\Gamma\left( j \right)\Gamma\left( \alpha-j+1 \right)}=-\frac{\alpha-j+1}{j}=1-\frac{\alpha+1}{j}$ .

# 6. Comparing the results of single fractional calculus terms and double fractional calculus terms, using the single chamber model as a benchmark

We compared the results of single fractional calculus terms and double fractional calculus terms under three ventilation modes, using M=N=5 as the benchmark. The corresponding results for BIC, SSR, and runtime are as follows:

Table S6. Comparison of results between single fractional order term and double fractional calculus term models

| **Ventilation**  **modes** | **Model** | **PSO parameters** | **Order parameters** | **BIC** | **SSR** | **Time(s)** |
| --- | --- | --- | --- | --- | --- | --- |
| PCAC | **equation (21)** | N=5, M=5 | α=1.1347 | 1571.9530 | **1.1069** | 24.7259 |
|  | equation (22) | N=10, M=10 | α=0.8919,  β=4.1718 | 4466.0565 | 1.3425 | 110.9988 |
|  | equation (22) | N=20, M=20 | α=0.8571,  β=3.4297 | 4233.9401 | 1.3219 | 358.4521 |
|  | equation (22) | N=30, M=30 | α=0.8560,  β=2.8324 | 4232.6624 | **1.3218** | 1050.6086 |
| VCAC | **equation (21)** | N=5, M=5 | α=1.5369 | -2223.0448 | **0.8595** | 26.7856 |
|  | equation (22) | N=10, M=10 | α=0.7110,  β=1.8575 | -1240.2930 | 0.9177 | 66.3059 |
|  | equation (22) | N=20, M=20 | α=0.8896,  β=1.1396 | -1205.9370 | 0.9198 | 299.6149 |
|  | equation (22) | N=30, M=30 | α=0.8304,  β=2.2574 | -1316.5997 | **0.9130** | 508.0200 |
| VCSIMV | **equation (21)** | N=5, M=5 | α=1.5710 | 2887.3673 | **1.2084** | 27.9563 |
|  | equation (22) | N=10, M=10 | α=0.1824,  β=1.2364 | 3386.0668 | 1.2492 | 69.3197 |
|  | equation (22) | N=20, M=20 | α=0.2123,  β=1.1269 | 3182.4255 | 1.2324 | 370.7555 |
|  | equation (22) | N=30, M=30 | α=0.2097,  β=1.1045 | 3160.3550 | **1.2306** | 786.2970 |

* all calculation results are retained to four decimal places

As shown in the table, the number of fractional calculus terms does not significantly affect the BIC and SSR indicators, but the introduction of more parameters to be estimated significantly prolongs the calculation time. Therefore, the main text mainly recommends the model with a single fractional calculus term.

# 7. The double-exponential function grows faster than the factorial function

To prove that the growth rate of the double exponential function $f\left( x \right)=e^{e^{x}}$ is faster than the factorial function $f\left( x \right)=x!$, it is equivalent to proving:

$\lim_{x\to\infty} \frac{f\left( x \right)}{g\left( x \right)}=\lim_{x\to\infty} \frac{e^{e^{x}}}{x!}=\infty$ (7.1)

Firstly,

$x!=\prod_{k=1}^{x} k\leq\prod_{k=1}^{x} x=x^{x}$ (7.2)

Secondly

$\lim_{x\to\infty} \frac{xln\left( x \right)}{e^{x}}=\lim_{x\to\infty}e^{\left( \ln\left( x \right)+\ln\left( \ln\left( x \right) \right)-x \right)}=\lim_{x\to\infty}e^{\left( \frac{\ln\left( x \right)+\ln\left( \ln\left( x \right) \right)-x}{x} \right)x}=\lim_{x\to\infty}e^{(0+0-1)x}=0$ (7.3)

由于

$\lim_{x\to\infty}\left( e^{x}-xln\left( x \right) \right)=\lim_{x\to\infty}e^{x}(1-\frac{xln\left( x \right)}{e^{x}})$ (7.4)

Applying (7.3) to (7.4) gives:

$\lim_{x\to\infty}\left( e^{x}-xln\left( x \right) \right)=\lim_{x\to\infty}e^{x}\left( 1-0 \right)=\infty$ (7.5)

Then we have the following equation based on (7.2):

$\lim_{x\to\infty} \frac{e^{e^{x}}}{x!}\geq\lim_{x\to\infty} \frac{e^{e^{x}}}{x^{x}}=\lim_{x\to\infty}e^{{(e}^{x}-xln\left( x \right))}=\lim_{x\to\infty}e^{\infty}=\infty$ (7.6)

Therefore, (7.1) is proved.

# 8. Interpretation of empirical method for rapid estimation of fractional calculus and exponential term parameters

To expedite the parameter estimation of models containing fractional calculus and exponential terms in the main text, an empirical method is proposed here, which eliminates the simulation evaluation phase and directly calculates the model parameter estimates from the measured data (i.e., consistent with the computational instance results). Furthermore, based on the quickly obtained empirical parameter values, the range of parameter values searched by the PSO algorithm based on model simulation in the main text can be narrowed down, eliminating some invalid calculations to converge faster to a stable optimal solution. That is, for models containing fractional calculus terms, the method described in 8.1 below can be used for quick parameter estimation, and for models containing exponential terms, the method described in 8.2 below can be used for quick parameter estimation.

## 8.1 Quick parameter estimation for models containing fractional calculus terms

In this article, fractional calculus models are suitable for application in Pressure-Controlled ventilation mode, and the fractional calculus models proposed in the main text has a general form as follows:

$P=EV+RV^{'}+k_{1}\mathcal{D}_{t}^{\alpha_{1}}\left( V\left( t \right) \right)+k_{2}\mathcal{D}_{t}^{\alpha_{2}}\left( V\left( t \right) \right)+\cdots{+k}_{m}\mathcal{D}_{t}^{\alpha_{m}}\left( V\left( t \right) \right)+P_{0}$ (8.1.1)

For a certain fractional calculus term in it, its fractional order generating function can be set as:

$g_{p}^{\alpha}(z)={(g_{0}+g_{1}z+g_{2}z^{2}+\cdots+g_{p}z^{p})}^{\alpha}$ (8.1.2)

Its Taylor series expansion can be written as:

$g_{p}^{\alpha}(z)=\sum_{k=0}^{\infty} \omega_{k}z^{k}$ (8.1.3)

Replace the variable z in the above equation with $e^{-j\varphi}$, then we have:

$g_{p}^{\alpha}(e^{-j\varphi})=\sum_{k=0}^{\infty} \omega_{k}e^{-jk\varphi}$ (8.1.4)

In the form of the Fourier Transform, each term $\omega$ is the Fourier coefficient of the function $g_{p}^{\alpha}(e^{-j\varphi})$, and each coefficient can be obtained through the Fast Fourier Transform (FFT) algorithm, reflecting the relationship between fractional calculus and Fourier Transform.

Different fractional orders can be compared to different frequency sine and cosine function terms, and coefficients such as k1, k2 in equation (8.1.1) can be analogous to corresponding weights. For the simplified single fractional order calculus model, i.e., equation (21) or the mixed model equation (33), in a series of test calculations, when the optimal order is found, its coefficient is often a higher value in the nearby area, which is equivalent to finding the most important component in the target P-V relationship. Based on this assumption, the search for the most suitable order can be based on the higher value of the fractional order term coefficient obtained from equation (37) as an indicator for continuous iterative calculation. Similarly, for multiple fractional order terms, searching for their optimal order is equivalent to finding the first principal component, the second principal component, etc. of the fitting target. The high coefficient terms found are retained as the optimized order, and the corresponding final overall model result is obtained in iterative calculation.

The optional approach can be based on the PSO algorithm. For a set of specified fractional orders, we can calculate the maximum value of the fractional order coefficients as the optimization target through equation (37) to obtain a stable optimal solution, which eliminates the need for simulation using the Simulink method, speeding up computation. It is not necessary to calculate SSR and RMSE for each round of simulation, and the optimization results can be quickly obtained.

## 8.2 Quick parameter estimation for models containing exponential terms

The exponential model is applicable in describing the step characteristics in the respiratory system based on the calculation results of measured data. For the exponential terms in equations (30-32) or in the mixed model equation (33), since each specified x value can obtain other undetermined parameter values based on equation (37), that is, the other parameters of the exponential model depend on the x value, then such models can be represented as:

$P\left( t \right)=f_{exp}(x,V(t))$ (8.2.1)

For a more intuitive explanation, we will use the numerical calculation results of equation (31) in the VC-AC ventilation mode in Table 2 of the main text as a specific example. Based on the data of case1VCAC1.txt, the curve of the exponential term parameters “x” and “a” estimated by equation (31) changes with respiratory volume V(t) as follows:


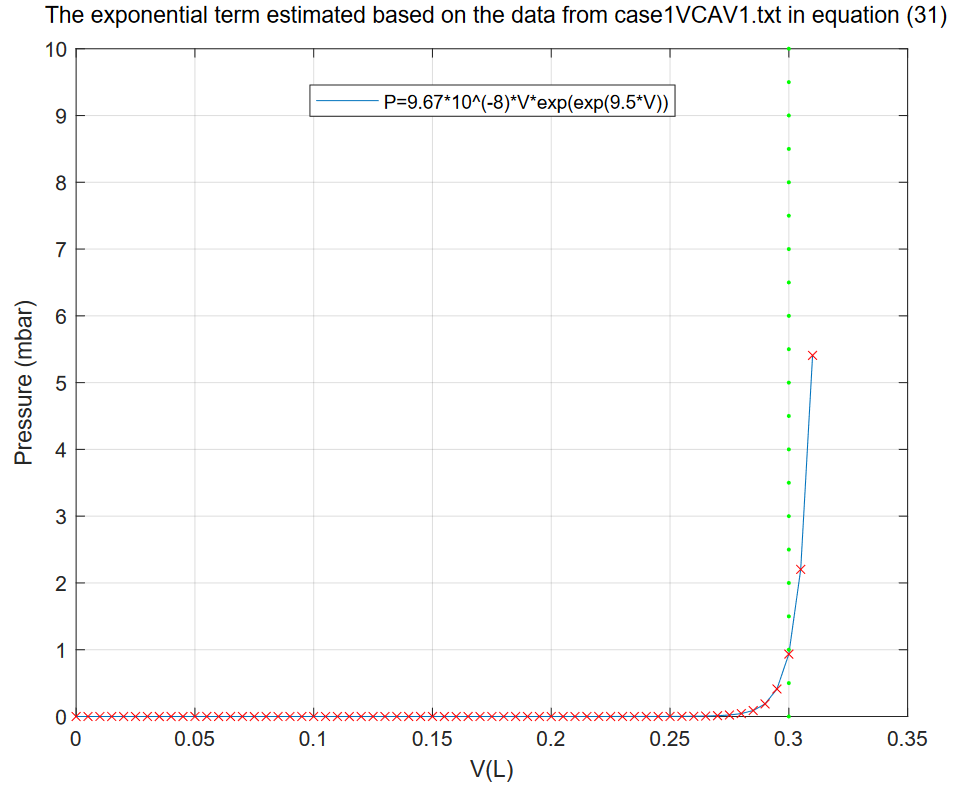


Supplementary Figure 8.2.1 The exponential term estimated based on the data from case1VCAV1.txt in equation (31). The green dashed line in the figure shows that the tidal volume set under this ventilation mode is 0.3L.

From this result, we can see that in the VC-AC mode, equation (31) undergoes a step change when respiratory volume V(t) reaches the preset tidal volume V_T_=0.3L (or corresponding to the total lung capacity, TLC, in spontaneous breathing). That is, it has the following characteristics:

$\left\{ \begin{matrix} \lim_{V\left( t \right)\to V_{T}-\Delta V} f_{exp}(x,V(t)) \to0 \\ \lim_{V\left( t \right)\to V_{T}+\Delta V} f_{exp}(x,V(t)) \to\infty\end{matrix} \right.$ (8.2.2)

Further, we take the time period from 0 to 5 seconds as an example to show the change of respiratory volume V(t) with pressure P(t) when V(t) reaches the preset tidal volume, as shown in the following figure:


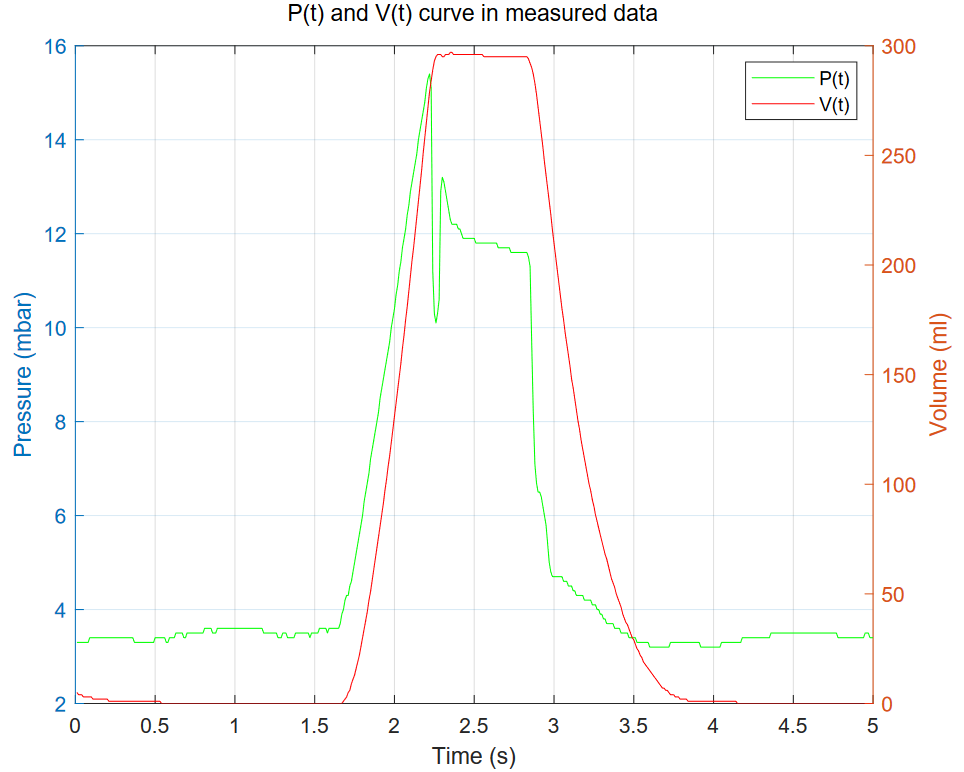


Supplementary Figure 8.2.2. P(t) and V(t) curve in measured data. The vertical axis on the left of the graph represents pressure P(t), the vertical axis on the right represents respiratory volume V(t), and the horizontal axis represents time in seconds.

The green and red lines in supplementary figure 8.2.2 respectively show the curves of the measured pressure P(t) and respiratory volume V(t) in the time period of 0-5s. When V(t) reaches the preset tidal volume stage, P(t) is the plateau pressure (Pplat) stage, that is:

$f_{exp}\left( x,V_{T} \right)=Pplat$ (8.2.3)

In the above formula, V_T_ and Pplat are known values, the unknown parameter x can be obtained from equation (8.2.3) and equation (37).

For situations where it is difficult to directly obtain analytical solutions for transcendental equations, we can iteratively obtain numerical solutions based on the PSO algorithm. This method eliminates the need for simulation at each time point, significantly reducing the amount of computation and allowing for faster estimation of exponential model parameters. Compared to the method of parameter estimation after simulation calculations in the main text, this empirical estimation may reduce accuracy while speeding up computation.

Here, one single exponential term corresponds to one step characteristic in the respiratory system. If multiple step changes appear in the respiratory volume V(t) curve in a certain respiratory system (which is rare), it generally corresponds to an equal number of exponential terms. At the same time, in a specific respiratory system, there will generally be an equal number of constraints in the form similar to equation (8.2.3), which can be used for further rapid solution of undetermined parameters. If the Pressure-Controlled ventilation mode primarily reflects the mechanical characteristics of resistance and inertia in the respiratory system, and the step characteristic of elasticity E is not significant, then after parameter estimation, the range of values for V(t) may all fall in the stable area of the exponential term (the value does not show significant changes), which is equivalent to the exponential term being invalid. For the model of equations (30-32), it is equivalent to degenerating and approximating to equation (1), and for the mixed model of equation (33), it is equivalent to degenerating and approximating to equation (21).

# 9. The Simulink model of different respiratory mechanics models

The main models involved in the main text that use Simulink model for simulation are the first-order differential model equation (1), the second-order model equation (10), the single-term fractional calculus model equation (21), the two-term fractional calculus model equation (22), the polynomial model equations (27-29), the exponential model equations (30-32), and the mixed model equation (33). The corresponding Simulink models are shown below:


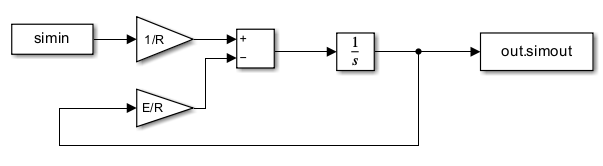


Supplementary Figure 9.1. Simulink model of first order linear differential model equation (1). The names of parameters in the figure are consistent with the naming of parameters in equation (1).


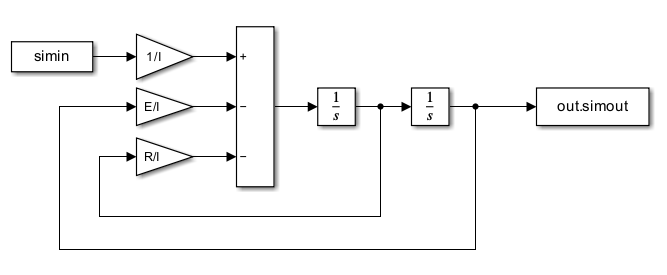


Supplementary Figure 9.2. Simulink model of second order linear differential model equation (10).


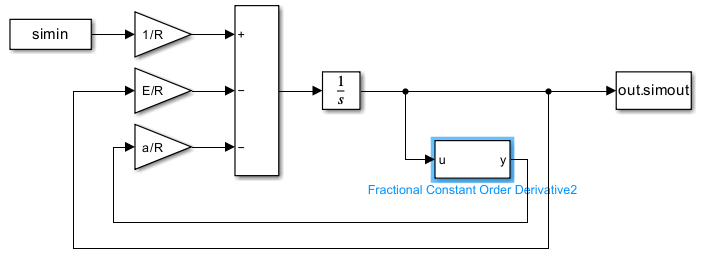


Supplementary Figure 9.3. Simulink model of fractional calculus model equation (21). The blue module in the figure is a fractional calculus simulation item based on the open-source vfoderiv3.


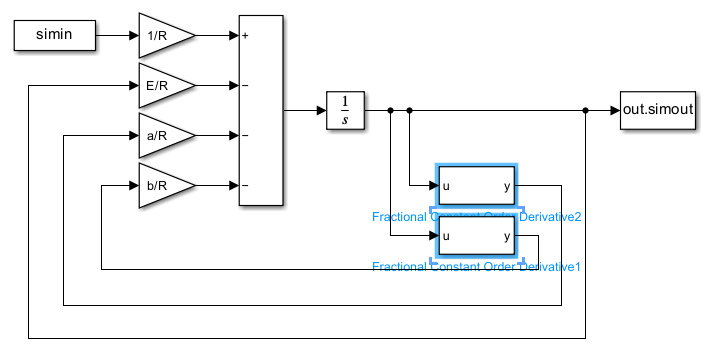


Supplementary Figure 9.4. Simulink model of fractional calculus model equation (22).


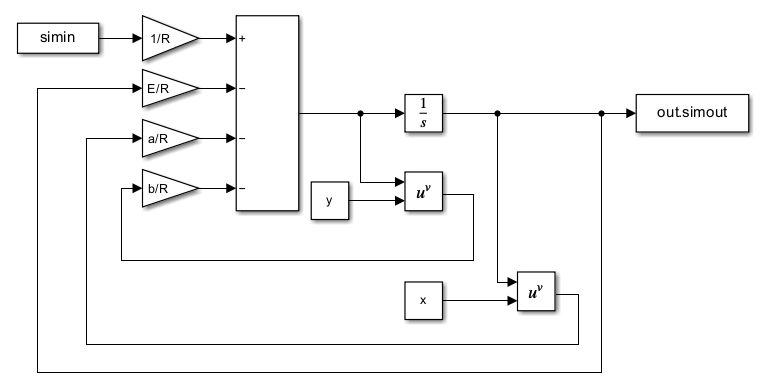


Supplementary Figure 9.5. Simulink model of polynomial model equation (27).


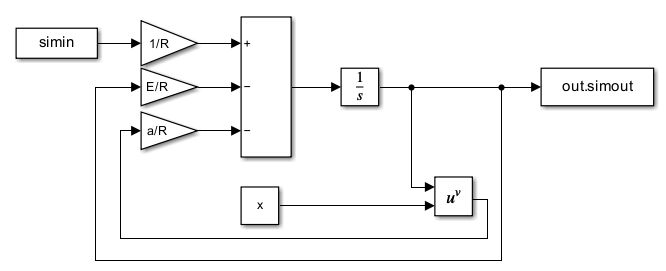


Supplementary Figure 9.6. Simulink model of polynomial model equation (28).


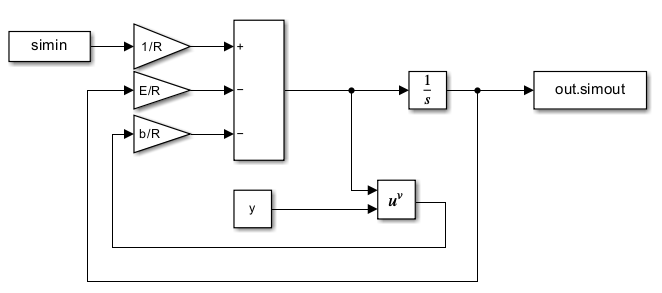


Supplementary Figure 9.7. Simulink model of polynomial model equation (29).


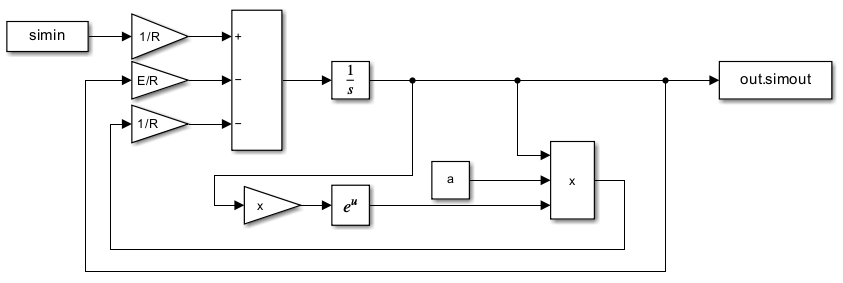


Supplementary Figure 9.8. Simulink model of exponential model equation (30).


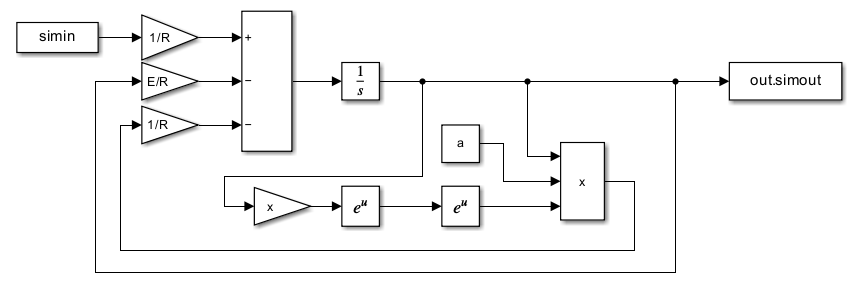


Supplementary Figure 9.9. Simulink model of exponential model equation (31).


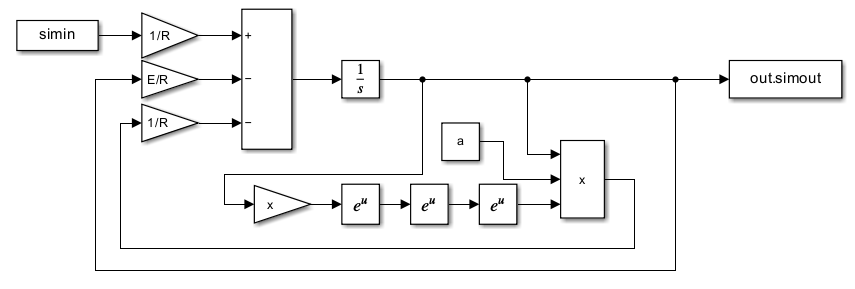


Supplementary Figure 9.10. Simulink model of exponential model equation (32).


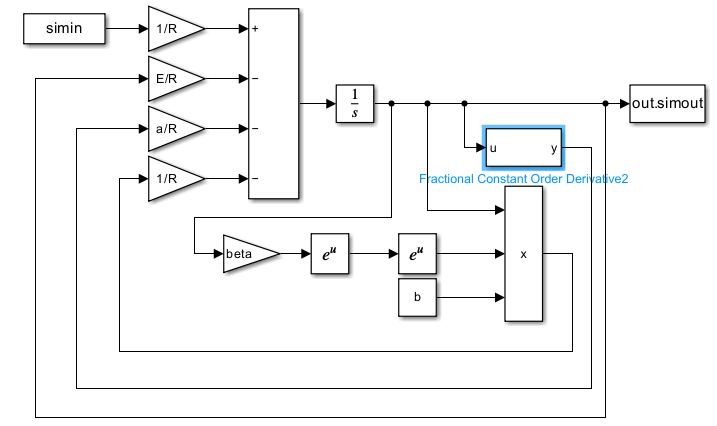


Supplementary Figure 9.11. Simulink model of mix model equation (33).

# 10. Derivation of the calculation formula for equivalent BIC

The original definition of BIC is^[2]^：

$\mathrm{BIC}=-2\ln\left( L \right)+k*ln(n)$ (10.1)

where L is the value of the likelihood function, and k is the number of model parameters.

If we assume that the residuals $\varepsilon$ follow a normal distribution, that is,

$V_{i}-\hat{V_{i}}=\varepsilon=\sim N(0,\sigma^{2})$ (10.2)

where $V_{i}$ is the actual measured respiratory volume, and $\hat{V_{i}}$ is the predicted volume from the respiratory mechanics model.

The likelihood function $L\left( \theta| \mathrm{data} \right)$ with respect to the parameter $\theta$ numerically equals the probability of the occurrence of the variable data given the parameter $\theta$:

$L\left( \theta| data \right)=p\left( data | \theta\right)=\prod_{i=1}^{N} p(\varepsilon_{i}|\theta)=\prod_{i=1}^{N} \frac{1}{\sqrt{2\pi}\sigma}exp(-\frac{{\varepsilon_{i}}^{2}}{2\sigma^{2}})$ (10.3)

The log-likelihood function is

$ln(L)=\sum_{i=1}^{N} (-\frac{1}{2}\ln\left( 2\pi\right)-\frac{1}{2}\ln\left( \sigma^{2} \right)-\frac{1}{2}(\frac{{\varepsilon_{i}}^{2}}{\sigma^{2}}))$ (10.4)

Substituting $\sigma^{2}=\frac{\mathrm{SSR}}{n}$, we get:

$ln(L)=-\frac{n}{2}\ln\left( 2\pi\right)-\frac{n}{2}\ln\left( \frac{\mathrm{SSR}}{n} \right)-\frac{n}{2}$ (10.5)

Substituting equation (10.5) into equation (10.1) gives:

$BIC=n\ln\left( 2\pi\right)+n\ln\left( \frac{\mathrm{SSR}}{n} \right)+n+k*ln\left( n \right)$ (10.6)

After removing irrelevant constants, we have:

$BIC=nln(SSR)+k*ln(n)$ (10.7)

# 11. The linear fitting results of the SSR values of the polynomial class model.

Based on the SSR values of the polynomial type model equations (27-29) through orders 1-11, for each x value, we calculate the mean and the logarithm of the mean of the SSR values corresponding to different y values, and obtain the following table of linear regression analysis results of SSR values with respect to x.

Table s11 linear regression analysis results in three ventilation modes data

| **Ventilation mode data** | **Type** | **Coefficient of  determination r2** | **P-value** |
| --- | --- | --- | --- |
| case1PCAC1.txt | mean of SSR | 0.8513 | 5.2112E-05 |
|  | logarithm of the mean of SSR | **0.8908** | **1.2724E-05** |
| case1VCAC1.txt | mean of SSR | 0.9061 | 6.4015E-06 |
|  | logarithm of the mean of SSR | **0.9204** | **3.0293E-06** |
| case1VCSIMV1.txt | mean of SSR | 0.9324 | 1.4457E-06 |
|  | logarithm of the mean of SSR | **0.9518** | **3.1347E-07** |

* all calculation results are retained to four decimal places

From the results in the table, it can be seen that, compared to the mean SSR value, the logarithm of the mean SSR value shows a more obvious linear correlation with x. That is, polynomial type model exhibits an approximate negative exponential trend as the exponent x increases.

# 12. Results of different respiratory mechanical models under Airway Pressure Release Ventilation (APRV) ventilation mode.

Airway Pressure Release Ventilation (APRV), is a type of pressure-controlled partial assist ventilation, typically used for rescue or replacement in patients with acute respiratory distress syndrome (ARDS) and hypoxemia who are difficult to tolerate conventional mechanical ventilation. In this section, measured respiratory mechanics data from a new type of APRV ventilation mode is selected as supplementary validation.

A new Bama pig (id as case4, weight 21.5kg) was prepared for APRV ventilation study. Consistent with the tidal volume in section 2.2, we use Dräger Savina 300 to measure airway pressure P(t), flow speed V’(t) and respiratory volume V(t) in the actual respiration of case4 Bama pig. The corresponding pressure parameters are set to keep the tidal volume at about 0.3L.

Table s12 linear regression analysis results in three ventilation modes data

| **Ventilation modes** | **Model** | **BIC** | **SSR** | **RMSE** | **Time（s）** |
| --- | --- | --- | --- | --- | --- |
| APRV | equation (1) | 2935.4332 | 1.2138 | 0.0090 | 0.197786 |
|  | equation (21) | 2296.3837 | 1.1617 | 0.0088 | 23.087733 |
|  | equation (31) | -7755.9444 | 0.59436 | 0.0063 | 23.369165 |
|  | equation (33) | **-8371.6219** | **0.56973** | **0.0062** | 320.43379 |

The data results are consistent with the main conclusions in the main text. That is, all models can well describe the APRV ventilation mode, and from the perspective of SSR and BIC values, the hybrid model is the most suitable.

# 13. Computational platform

Numerical results have been obtained on an ordinary personal computer with an Intel(R) Core (TM) i7-1165G7 @ 2.80GHz CPU and 16GB of memory. In order to compare performance in the same conditions, all the calculations have been performed using single-thread computing.

# Reference

[1] Dincel E. Advanced mechanical ventilation modes: design and computer simulations. Comput Methods Biomech Biomed Engin. 2021 May;24(6):673-686.

[2] Neath, A. A. & Cavanaugh, J. E. The Bayesian information criterion: background, derivation, and applications. WIREs Comp Stat 4, 199–203 (2012).
